# Deepfake face detection using hybrid bag-of-visual-words and multi-CNN feature fusion

**DOI:** 10.1038/s41598-026-53464-w

**Published:** 2026-05-19

**Authors:** Maher Alrahhal, Fatimah Alqahtani, Rohaya Latip, Mohammad AlShabi, Walaa M. Abd-Elhafiez

**Affiliations:** 1 Research Institute of Sciences and Engineering, University of Sharjah, Sharjah, UAE; 2 Department of Computer Science, College of Engineering and Computer Science, Jazan University, Jazan, Kingdom of Saudi Arabia; 3 Faculty of Computer Science and Information Technology, University Putra Malaysia, Serdang, Malaysia; 4 Department of Mechanical and Nuclear Engineering, College of Engineering, University of Sharjah, Sharjah, UAE; 5 Department of Mechanical Engineering, McMaster UniversityCanada, Hamilton, Canada; 6 Department of Computer Science, Faculty of Computers and Artificial Intelligence, Sohag University, Sohag, Egypt

**Keywords:** Deepfake detection, Forensic AI, Feature fusion, Bag-of-visual-words (BoVW), HOG, Transfer learning, SVM, Engineering, Mathematics and computing

## Abstract

Recent advances in generative modeling have enabled the creation of highly realistic deepfake facial images, posing significant risks to digital security, media integrity, and public trust. Although deep learning–based detection methods have achieved strong performance, they often suffer from limited cross-dataset generalization, sensitivity to manipulation-specific artifacts, and reduced interpretability. To address these limitations, this paper proposes a forensic-first hybrid deepfake face detection framework that integrates handcrafted local forensic descriptors with multi-CNN deep semantic representations. Specifically, manipulation-sensitive regions are captured using a Bag-of-Visual-Words (BoVW) model constructed from Histogram of Oriented Gradients (HOG) features extracted at salient keypoints detected via SURF, FAST, and BRISK. In parallel, high-level features are obtained from fine-tuned ResNet-50, MobileNet, and ShuffleNet models and fused at the feature level to capture complementary semantic information. The combined feature representation is classified using a Support Vector Machine (SVM), enabling stable decision boundaries and improved generalization. Extensive experiments on six benchmark datasets of varying scale and complexity demonstrate that the proposed approach consistently outperforms state-of-the-art methods, achieving up to 97.55% accuracy while maintaining robustness under cross-dataset and challenging forensic conditions. The results highlight the effectiveness of integrating explicit forensic features with deep representations to achieve a robust, interpretable, and generalizable solution for deepfake face detection.

## Introduction

Recent advances in deep learning have significantly accelerated progress in generative modeling, particularly through Generative Adversarial Networks (GANs)^1^. While these models have enabled beneficial applications in digital entertainment, content synthesis, and virtual reality, they have also facilitated the creation of highly realistic forged images and videos, commonly referred to as deepfakes. By manipulating facial identity, expressions, or visual appearance with remarkable fidelity, deepfake technology poses serious threats to digital security, privacy, and societal trust. Its misuse has been increasingly reported in political misinformation campaigns, identity impersonation, financial fraud, and the dissemination of non-consensual content, raising urgent concerns regarding media authenticity and forensic reliability^2^.

Detecting deepfake media has become progressively more challenging as generative models continue to evolve. Early detection approaches primarily relied on identifying visual artifacts or statistical inconsistencies introduced during the synthesis process, such as unnatural textures, blending boundaries, or color distortions^3^. However, modern GAN architectures are explicitly designed to suppress these artifacts, producing outputs that are often visually indistinguishable from authentic media. Consequently, traditional forensic methods that depend solely on handcrafted or shallow statistical features struggle to generalize to newly emerging manipulation techniques and unseen forgery patterns^4^.

The challenge is further exacerbated when manipulated images undergo common post-processing operations, including JPEG compression, resizing, gamma correction, and noise injection. Such operations may attenuate manipulation traces while simultaneously introducing new artifacts that confuse detection algorithms, thereby reducing robustness and reliability in real-world deployment scenarios^5^. Effective deepfake detection systems must therefore identify extremely subtle manipulation cues while maintaining robustness under diverse image transformations and evolving forgery strategies.

To address these challenges, recent research has increasingly focused on deep learning–based detection frameworks, particularly Convolutional Neural Networks (CNNs), due to their ability to learn hierarchical semantic representations directly from data. CNN-based models have demonstrated promising performance in deepfake detection by capturing complex visual patterns and facial semantics^4^. Nevertheless, purely deep learning–driven approaches often suffer from limited cross-dataset generalization, sensitivity to domain shifts, and overfitting to specific manipulation techniques or training distributions^6,7^. Moreover, end-to-end CNN architectures tend to prioritize high-level semantic cues, potentially overlooking fine-grained local inconsistencies that may serve as critical forensic evidence in subtle facial manipulations.

In contrast, handcrafted feature descriptors—such as Histogram of Oriented Gradients (HOG) and Bag-of-Visual-Words (BoVW) representations^8,9^—are effective in capturing local texture, edge orientation, and structural information that can reveal manipulation artifacts at a micro-level^10,11^. These descriptors offer robustness, interpretability, and resilience to certain image transformations. However, when used independently, they lack the representational capacity required to model the complex semantic variations present in modern deepfake content, limiting their effectiveness as standalone detectors.

Motivated by the complementary strengths and limitations of deep learning–based and handcrafted feature approaches, this work proposes a forensic-first hybrid deepfake face detection framework that explicitly integrates local forensic descriptors with fused deep CNN representations. Unlike existing deep-first or end-to-end hybrid approaches, the proposed framework structurally prioritizes **explicit local forensic evidence** and employs deep models as complementary semantic encoders rather than sole decision-makers.

Specifically, local interest points are extracted using multiple complementary detectors—SURF^12^, FAST corner detection^13^, and BRISK^14^—to explicitly localize facial regions that are more susceptible to manipulation artifacts. The most salient keypoints are retained and described using HOG features^11^, which are subsequently clustered via the K-means + + algorithm^15^ to construct a discriminative BoVW representation. In parallel, high-level semantic features are extracted from multiple pretrained CNN architectures—ResNet-50^16^, MobileNet^17^, and ShuffleNet^18^—each fine-tuned through transfer learning to capture complementary deep representations. Handcrafted and deep features are fused at the feature level and classified using a Support Vector Machine (SVM)^19^, enabling stable decision boundaries and improved generalization under cross-dataset evaluation.

The main contributions of this study are summarized as follows:

- *Forensic-First Hybrid Detection Framework*: This work introduces a forensic-first deepfake face detection framework that explicitly prioritizes handcrafted local forensic descriptors and integrates them with deep semantic features, differing fundamentally from end-to-end deep or deep-only hybrid approaches.
- *Explicit Manipulation-Sensitive Localization*: A multi-detector interest point strategy (SURF, FAST, and BRISK) is employed to explicitly localize facial regions prone to manipulation artifacts, improving robustness to pose variation, partial facial visibility, and localized forgeries.
- *BoVW–HOG Representation for Deepfake Forensics*: A BoVW representation constructed from HOG descriptors is tailored for deepfake detection, enabling stable modeling of subtle gradient and texture inconsistencies that are often suppressed by deep networks.
- *Decoupled Multi-CNN Feature Fusion Strategy*: Deep features from heterogeneous CNN architectures are fused at the feature level and decoupled from classification using an SVM, reducing overfitting and improving cross-dataset generalization.

The proposed framework is designed to address the following research questions:

The proposed framework is designed to address the following key research questions:

- RQ1: Can the integration of handcrafted forensic descriptors with deep CNN features improve deepfake face detection performance compared to single-representation approaches?
- RQ2: How effectively does the proposed hybrid framework generalize across datasets of varying scale, quality, and manipulation complexity?
- RQ3: What is the impact of multi-CNN feature fusion on capturing complementary semantic information and enhancing detection robustness?
- RQ4: How does the use of an SVM classifier influence model stability, interpretability, and generalization compared to end-to-end deep learning classifiers?

Extensive experimental evaluations conducted across multiple benchmark datasets—including the 130 K Real vs. Fake Face dataset^20^, Real vs. AI-Generated Faces^21^, Real/Fake Cropped Faces^22^, RVF10K^23^, Fake vs. Real Faces (Hard)^24^, and the Human Faces dataset^25^—demonstrate that the proposed method consistently outperforms CNN-only and handcrafted-feature-based approaches, confirming its robustness and generalization capability.

The remainder of this paper is organized as follows. Section II reviews related work in deepfake detection and digital image forensics. Section III describes the proposed hybrid detection framework in detail. Section IV presents experimental results and comparative evaluations against state-of-the-art methods. Finally, Section V concludes the paper and outlines directions for future research.

## Related work

Deepfake face detection has rapidly evolved into a multidisciplinary research field encompassing convolutional neural networks, transformer-based architectures, frequency-domain analysis, spatiotemporal modeling, hybrid feature fusion, and explainable artificial intelligence. This section reviews the most relevant studies by grouping them into major methodological categories, as summarized in Table 1, and highlights their limitations to motivate the proposed approach.

### CNN-based deepfake face detection

Early deepfake detection research primarily relied on CNNs to extract discriminative spatial features from facial images. These approaches exploit the strong representation capability of CNNs to capture local texture inconsistencies, edge irregularities, and structural distortions introduced during face manipulation. A wide range of studies employed single or fused CNN backbones such as ResNet, DenseNet, EfficientNet, MobileNet, and Inception architectures to identify manipulation artifacts in facial textures and structures. For example, Devi and Ben Sujitha^26^ proposed a hybrid DenseNet-121 and Inception-V3 model to improve feature diversity and representation capacity, while Bapatla and Shashi^27^ combined DCGAN-based sample generation with ResNet50 classification to enhance detection performance by exposing the classifier to diverse forged patterns.

Similarly, AlRowais et al.^28^ introduced a deep feature fusion framework using EfficientNet and ShuffleNet, followed by an Extreme Learning Machine classifier, targeting deepfake detection in consumer-space environments. These CNN-based approaches generally demonstrate strong performance under controlled experimental conditions and within-dataset evaluation, benefiting from transfer learning and large-scale pretraining on natural image datasets.

However, despite their effectiveness, CNN-based detectors often exhibit limited cross-dataset generalization. This limitation arises because CNNs tend to overfit manipulation-specific artifacts, dataset-dependent biases, and RGB-domain cues that may not persist across different forgery generation methods or post-processing operations such as compression, resizing, and noise injection. Moreover, deep CNNs primarily focus on high-level semantic representations, which may suppress subtle local forensic traces crucial for distinguishing highly realistic forgeries. These challenges motivate the exploration of complementary feature representations and hybrid detection strategies that can enhance robustness and generalization beyond purely CNN-driven models.

### Transformer-based and attention-driven methods

To address the limited receptive field and locality bias of conventional CNNs, transformer-based architectures have been increasingly adopted for face forgery detection. Vision Transformers (ViTs) leverage self-attention mechanisms to model long-range dependencies and capture global inconsistencies that may arise across different facial regions in manipulated images. By treating an image as a sequence of patches, ViTs enable the modeling of inter-patch relationships, which is particularly beneficial for detecting subtle global artifacts that are difficult to identify using convolutional operations alone. Ambreen et al.^29^ proposed a patch-based ViT–CNN hybrid model (PViT), demonstrating that combining CNN-based local feature extraction with transformer-based global reasoning significantly improves detection accuracy and robustness across large-scale datasets.

Building upon basic ViT architectures, more advanced transformer designs have been introduced to further enhance representation capability and generalization. Zhang et al.^30^ presented a distilled transformer network with locally enhanced global representations, incorporating mixture-of-experts learning and self-distillation to mitigate attention collapse and improve cross-manipulation robustness. Similarly, Zheng et al.^31^ proposed FETNet, a frequency-enhanced transformer network that integrates RGB and frequency-domain information through learnable frequency enhancement modules and global transformer-based fusion. These approaches demonstrate that attention mechanisms and transformer architectures can effectively capture both global context and frequency-related forgery cues.

Despite their promising performance, transformer-based and attention-driven methods introduce several practical challenges. These models typically require large-scale training data, extensive computational resources, and careful hyperparameter tuning to stabilize attention learning. Furthermore, their high architectural complexity often reduces interpretability, making forensic analysis and explainability difficult—an important requirement in legal and security-critical applications. Additionally, transformer-based models may suffer from increased inference latency, limiting their applicability in real-time or resource-constrained forensic systems. These limitations highlight the need for alternative hybrid frameworks that retain strong representational power while improving efficiency, interpretability, and robustness.

### Frequency-domain and texture-based detection

Another prominent line of research exploits frequency-domain inconsistencies and texture irregularities introduced during face manipulation. Unlike RGB-domain analysis, frequency-based methods reveal subtle artifacts arising from resampling, blending, and synthesis operations that are often imperceptible to the human eye. Techniques based on the Discrete Cosine Transform (DCT), wavelet decomposition, Fourier spectrum analysis, and high-frequency residual modeling have demonstrated that forged facial images typically exhibit abnormal frequency distributions and texture distortions.

Wang et al.^32^ proposed the WATCHER framework, which leverages wavelet-guided hierarchical texture–content relation learning to capture fine-grained forgery cues while enhancing cross-dataset generalization. By explicitly modeling texture–content interactions through wavelet-based representations, their method addresses overfitting to manipulation-specific patterns. Similarly, Gong et al.^33^ and Zhou et al.^34^ integrated spatial and frequency-domain features using attention-based fusion mechanisms, enabling the detector to jointly reason about global facial structure and frequency-domain anomalies. These approaches demonstrated improved robustness against post-processing operations such as compression and resizing, which often degrade purely spatial detectors.

Uddin et al.^35^ further advanced this direction by introducing multi-scale cross-attention mechanisms to fuse spatial and frequency representations, allowing the model to adaptively emphasize discriminative cues across scales. While these frequency-domain and texture-based approaches substantially improve detection robustness and generalization, they typically rely on complex deep architectures with multiple attention modules and frequency transformation stages. This increased architectural complexity can hinder interpretability, elevate computational cost, and limit deployment in real-time or resource-constrained forensic environments. Consequently, there remains a need for hybrid detection strategies that preserve the discriminative power of texture and frequency cues while maintaining computational efficiency and transparency.

### Spatiotemporal and video-based detection

Video-based deepfake detection methods exploit temporal inconsistencies across consecutive frames, leveraging the fact that many manipulation techniques introduce subtle temporal artifacts in facial motion, expressions, and physiological signals. These approaches typically integrate spatial feature extraction with sequential modeling to capture inconsistencies that may not be observable in isolated frames. Recurrent neural networks (RNNs), Long Short-Term Memory (LSTM) networks, and more recently, state-space models have been widely adopted for this purpose.

Ansar and Kumar^36^ proposed a hybrid framework that combines time-distributed MobileNetV2 with an LSTM network to model motion irregularities across video frames, demonstrating improved robustness over purely frame-based CNN detectors. Similarly, Rahman et al.^37^ introduced DeepJurist, a forensic-oriented architecture integrating CNN-based spatial encoding, Bi-LSTM temporal modeling, and multi-head attention to enhance temporal dependency learning in judicial video evidence. Their approach highlights the importance of temporal coherence analysis in high-stakes applications such as legal proceedings.

More recently, Chen et al.^38^ advanced spatiotemporal modeling by introducing a dual-stream framework based on phase-consistent edge features and 3D visual state-space representations, enabling the capture of both spatial forgery traces and long-range temporal dynamics. By leveraging modern state-space modeling techniques, their method achieves strong cross-dataset generalization and robustness to post-processing perturbations.

Despite their effectiveness, spatiotemporal and video-based detection methods are inherently computationally intensive, requiring frame-level preprocessing, temporal alignment, and sequential inference. Moreover, their reliance on video data limits applicability in many real-world forensic scenarios where only still images are available, such as social media profile images, identity documents, and static news imagery. These constraints motivate the development of image-level detection frameworks that can achieve high accuracy and robustness without temporal information, while maintaining lower computational cost and broader applicability.

### Hybrid feature fusion and graph-based models

To overcome the generalization limitations of single-model architectures, recent research has increasingly focused on hybrid feature fusion strategies that integrate complementary representations from multiple deep learning streams. Gong et al.^33^ proposed a multi-scale and multi-domain fusion framework that jointly models spatial and frequency-domain cues through attention-guided feature interaction, demonstrating improved robustness under compression and cross-dataset evaluation. Similarly, Duan et al.^39^ introduced a two-stream, multi-scale enhancement network that encourages the model to learn generic forgery features by overlapping forged and real feature distributions, achieving stronger generalization performance across unseen datasets.

In an effort to balance accuracy and efficiency, Ghosh and Naskar^40^ presented a multi-level feature fusion (MLFF) framework that integrates representations across different network depths using lightweight backbones. Their approach captures artifacts at multiple resolutions while significantly reducing model complexity, making it suitable for resource-constrained environments. Although these fusion-based methods improve robustness compared to single-stream CNNs, they remain entirely dependent on deep representations and require careful architectural design and extensive training to stabilize multi-stream interactions.

Parallel to fusion-based strategies, graph-based deepfake detection models have emerged as a promising direction for capturing relational inconsistencies among facial regions. Su et al.^41^ modeled facial components as nodes in a graph structure and employed graph attention networks (GATs) to learn intrinsic relationships between regions, thereby avoiding rigid grid-based image representations. Yu et al.^42^ further introduced a feature disentangling and multi-view learning framework that explicitly separates forgery-relevant features from source-dependent noise, improving robustness against dataset bias and manipulation-specific artifacts.

Despite their effectiveness, both fusion-based and graph-based approaches remain fully deep learning–driven, relying exclusively on learned representations. They lack explicit handcrafted forensic descriptors that encode well-established image statistics, local gradient structures, or keypoint-based irregularities. As a result, these models often suffer from reduced interpretability and may still overfit subtle dataset-specific cues. This gap motivates hybrid frameworks that integrate handcrafted local descriptors with deep semantic features, enabling complementary learning and more transparent forensic reasoning.

### Explainability in deepfake detection

With the increasing deployment of deepfake detection systems in sensitive domains such as digital forensics, social media moderation, and judicial decision-making, explainability and transparency have become critical requirements. Understanding *why* a model classifies an image as real or fake is essential for trust, accountability, and legal admissibility. In this context, Alotaibi^43^ provided a comprehensive survey of explainable AI (XAI) techniques applied to deepfake face detection, reviewing feature attribution methods, attention visualization, prototype-based explanations, and model-agnostic interpretability frameworks.

While these XAI techniques offer valuable post hoc insights into deep model decisions, they are typically applied after training and do not fundamentally alter the feature learning process. Consequently, they do not inherently improve robustness, generalization, or resistance to adversarial manipulation. Moreover, explanations derived from deep attention maps or gradient-based attribution may remain abstract and difficult to interpret from a forensic standpoint.

In contrast, detection frameworks that incorporate explicit handcrafted features, such as local keypoints, gradient distributions, and visual vocabularies, inherently provide more interpretable intermediate representations. These features are grounded in well-understood image statistics and forensic principles, enabling clearer reasoning about detected manipulations. This observation further motivates hybrid detection strategies that combine deep learning with handcrafted descriptors to jointly enhance accuracy, robustness, and explainability.

**Table 1:** Comparative analysis of related deepfake face detection approaches.

Category	Representative works	Core methodology	Strengths	Key limitations	Gap addressed by our work
CNN-Based Spatial Methods	Devi & Ben Sujitha^26^; Bapatla & Shashi^27^; AlRowais et al.^28^	Single and fused CNN backbones (DenseNet-121, Inception-V3, ResNet50, EfficientNet, ShuffleNet)	Strong spatial feature learning; high within-dataset accuracy; effective transfer learning	Limited cross-dataset generalization; overfitting to RGB-domain artifacts; weak interpretability	We fuse CNN features with BoVW–HOG handcrafted descriptors, reducing dataset bias and improving robustness
Transformer & Attention-Driven Models	Ambreen et al.^29^; Zhang et al.^30^; Zheng et al.^31^	ViT–CNN hybrids, distilled transformers, frequency-enhanced attention fusion	Capture long-range dependencies; improved global reasoning; stronger robustness	High computational cost; complex training; reduced transparency; higher inference latency	Our approach avoids transformers, achieving competitive robustness with lower complexity and clearer feature semantics
Frequency-Domain & Texture-Based Methods	Wang et al. (WATCHER)^32^; Gong et al.^33^; Zhou et al.^34^; Uddin et al.^35^	Wavelet analysis, DCT/Fourier features, multi-scale frequency attention	Robust to compression, resizing, and post-processing; improved generalization	Deep frequency pipelines increase architectural complexity; limited interpretability	We capture texture irregularities using HOG and keypoint-based descriptors, offering a simpler and interpretable alternative
Spatiotemporal / Video-Based Models	Ansar & Kumar^3536^; Rahman et al.^37^; Chen et al.^38^	CNN + LSTM / Bi-LSTM; attention-based temporal modeling; state-space representations	Exploit temporal inconsistencies; strong performance on video deepfakes	Computationally expensive; video-only dependency; limited applicability to still images	Our framework is image-only, suitable for social media, identity documents, and static forensic analysis
Hybrid Deep Fusion Models	Gong et al.^3533^; Duan et al.^39^; Ghosh & Naskar^40^	Multi-stream, multi-scale deep feature fusion	Improved cross-dataset robustness compared to single-stream CNNs	Fully deep learning–driven; lacks explicit forensic descriptors; limited interpretability	We integrate deep + handcrafted features, improving generalization and forensic transparency
Graph-Based Detection Models	Su et al.^41^; Yu et al.^42^	Graph attention networks; relational modeling of facial regions; feature disentanglement	Capture inter-region relationships; mitigate rigid grid bias	High model complexity; abstract representations; difficult interpretability	Our method preserves explicit local structures (keypoints, gradients) without graph overhead
Explainable AI (XAI)	Alotaibi^43^	Post-hoc explanation (Grad-CAM, attribution, attention visualization)	Improves transparency and trust	Does not enhance robustness or feature learning; explanations remain abstract	Our handcrafted features provide intrinsic interpretability, not post-hoc explanations

### Research gap, motivation, and contribution of the proposed work

Despite substantial progress in deepfake face detection, several critical limitations remain unresolved in existing research. Most current approaches rely predominantly on deep learned representations, including CNNs, transformer-based models, and frequency-aware architectures^26,29,31^. While these methods demonstrate strong performance under constrained experimental settings, they often exhibit limited feature diversity, making them prone to overfitting manipulation-specific artifacts and reducing their ability to generalize to unseen or evolving deepfake generation techniques^30,33^. In contrast, explicit handcrafted forensic descriptors—such as local keypoints, gradient distributions, and visual vocabularies—remain largely underexplored in modern deepfake detection pipelines, despite their proven effectiveness in classical image forensics.

Furthermore, although hybrid deep fusion strategies have been proposed to enhance robustness, they typically integrate features within a single architectural paradigm or rely entirely on end-to-end deep learning^33,39^. Few studies systematically combine complementary deep representations from multiple CNN architectures with handcrafted local descriptors to jointly capture both high-level semantic information and low-level forensic cues. As a result, existing methods often struggle to simultaneously achieve robustness, cross-dataset generalization, and interpretability, as summarized in Table 1.

In addition, challenges related to generalization and transparency persist. Transformer-based and frequency-domain models improve robustness under certain conditions but introduce significant computational overhead and limited interpretability^31,32^. These drawbacks restrict their deployment in real-world forensic and legal scenarios, where efficiency, stability, and explainability are essential requirements^43^.

Motivated by these gaps, this work proposes a principled hybrid deepfake face detection framework that explicitly bridges handcrafted forensic features with deep learning representations. In contrast to CNN-, transformer-, or frequency-only approaches, the proposed method makes the following key contributions:

- *Handcrafted Forensic Feature Integration*: Histogram of Oriented Gradients (HOG) and a Bag-of-Visual-Words (BoVW) representation are employed to capture fine-grained local texture and structural inconsistencies that are often overlooked by purely deep models.
- *Robust Local Feature Extraction*: Multiple keypoint detectors are utilized to enhance robustness against pose variations, facial expression changes, and localized manipulations, increasing resilience to diverse forgery patterns.
- *Multi-CNN Feature Fusion*: Deep features extracted from multiple CNN architectures are fused with handcrafted descriptors at the feature level, rather than relying on monolithic end-to-end deep architectures, enabling complementary semantic–forensic representation learning.
- *Lightweight and Interpretable Classification*: A classical SVM classifier is adopted to improve interpretability, training stability, and generalization while reducing the risk of overfitting associated with deep classifiers.
- *Efficient and Generalizable Detection*: The proposed framework achieves strong cross-dataset performance with lower computational complexity, making it suitable for image-only deepfake detection in real-world forensic and educational applications.

By explicitly integrating handcrafted local forensic cues with deep semantic representations, the proposed approach offers a lightweight, interpretable, and generalizable alternative to existing deepfake detection systems. This unified strategy directly addresses key limitations related to robustness, transparency, scalability, and real-world applicability, positioning the proposed method as a practical and effective solution for modern deepfake face detection.

## Methodology

### Overview of the proposed framework

The proposed deepfake face detection framework is designed to effectively distinguish real and manipulated facial images by integrating handcrafted forensic descriptors with deep learning–based representations. The core motivation behind this design is to exploit the complementary strengths of low-level texture analysis and high-level semantic feature learning, thereby improving robustness, generalization, and interpretability.

**Fig. 1:**
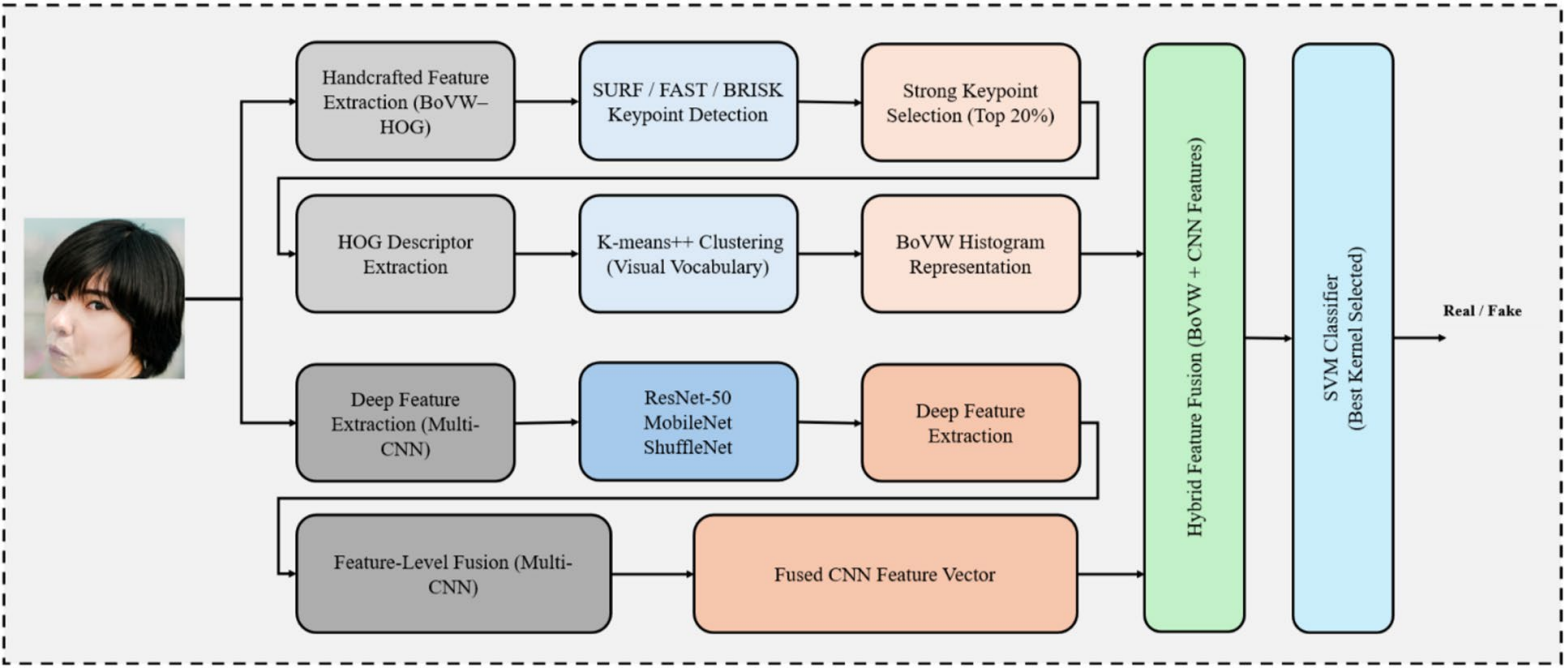
Overview of the proposed hybrid BoVW–HOG and multi-CNN feature fusion framework for deepfake face detection.

As illustrated in Fig. 1, the proposed framework consists of three main stages. In the first stage, handcrafted forensic features are extracted using a BoVW representation built upon HOG descriptors. Local interest points are detected using SURF, FAST corner detection, and BRISK algorithms, and only the most discriminative keypoints are retained to focus on regions that are more likely to contain manipulation artifacts. These descriptors are clustered using the K-means + + algorithm to construct a visual vocabulary, enabling each image to be represented as a histogram of visual word occurrences.

In the second stage, deep semantic features are extracted using multiple pre-trained CNN architectures, namely ResNet-50, MobileNet, and ShuffleNet. Each network is fine-tuned on the target dataset through transfer learning to adapt the learned representations to the deepfake detection task. Features extracted from selected high-level layers of each CNN are fused at the feature level to capture complementary spatial and semantic information while maintaining computational efficiency.

In the third stage, the handcrafted BoVW-HOG features and the fused CNN-based features are integrated to form a unified hybrid feature representation. This final feature vector is then used to train a SVM classifier, which provides stable decision boundaries and improved generalization compared to end-to-end deep classifiers. The trained SVM model outputs a binary decision indicating whether a given facial image is real or manipulated.

Overall, the proposed framework offers a lightweight yet powerful hybrid detection strategy that leverages both forensic texture cues and deep semantic representations. By combining classical computer vision techniques with modern deep learning models, the framework achieves high detection accuracy while remaining suitable for image-only forensic scenarios and real-world deployment.

### Handcrafted forensic feature extraction (BoVW–HOG Module)

**Fig. 2:**
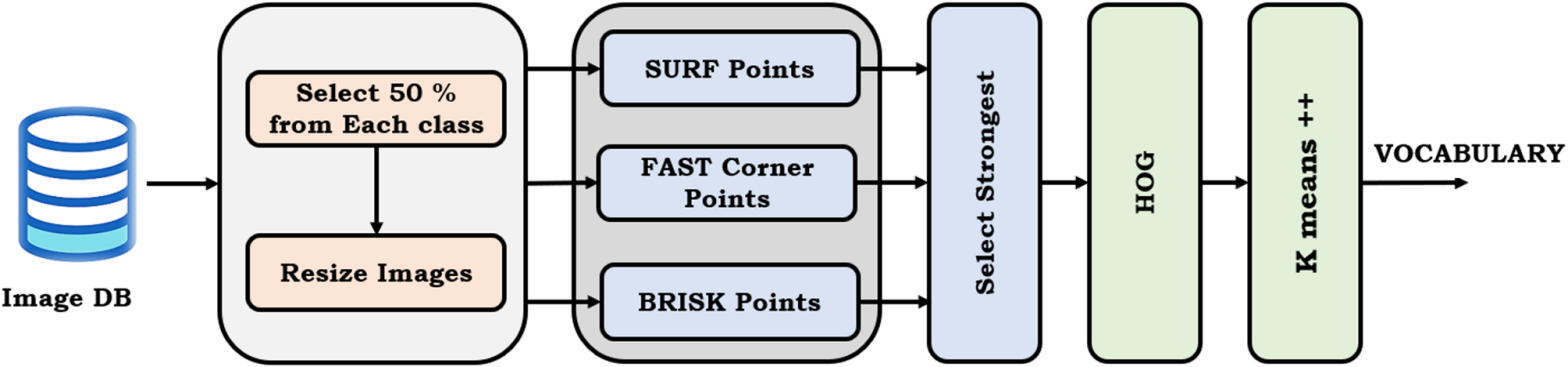
Handcrafted feature extraction pipeline using SURF/FAST/BRISK keypoints, HOG descriptors, and BoVW vocabulary construction.

The first stage of the proposed framework focuses on extracting handcrafted forensic features that capture fine-grained texture and structural inconsistencies commonly introduced during deepfake generation. This stage employs a BoVW^8,9^ representation built upon HOG descriptors^11,44,45^ and local interest point analysis. The overall workflow of this module is illustrated in Fig. 2.

#### Image selection and preprocessing

As shown in Fig. 2, a balanced subset comprising 50% of the images from each class (real and fake) is first selected from the image database to construct a representative visual vocabulary. All selected images are resized to a uniform spatial resolution to ensure consistency in subsequent feature extraction and descriptor computation^44,46^.

#### Local interest point detection

Local interest point detection is a critical component of the proposed deepfake face detection framework, as facial manipulations often introduce subtle, localized inconsistencies in texture, structure, and gradient distributions. To robustly capture such artifacts, a diverse set of complementary keypoint detectors is employed, including SURF, corner-based detectors (Harris, Shi–Tomasi, FAST), and BRISK. The joint use of multiple detectors improves robustness to pose variations, illumination changes, and localized manipulations.

SURF (Speeded-Up Robust Features) SURF^12,47^ is a scale- and rotation-invariant local feature detector and descriptor designed for efficient and robust keypoint extraction. As a detector, SURF approximates the Hessian matrix of the image intensity function. For a point $\:x=(x,y)$ at scale $\:\sigma\:$, the Hessian matrix is defined as

1 $$\:H(x,\sigma\:)=\left[\begin{array}{ll}{L}_{xx}(x,\sigma\:)&\:{L}_{xy}(x,\sigma\:)\\\:{L}_{xy}(x,\sigma\:)&\:{L}_{yy}(x,\sigma\:)\end{array}\right]$$

where $\:{L}_{xx},{L}_{yy}$, and $\:{L}_{xy}$ are second-order Gaussian derivatives. Interest points are detected by maximizing the determinant

2 $$\:\mathrm{d}\mathrm{e}\mathrm{t}\left(H\right)={L}_{xx}{L}_{yy}-{\left({L}_{xy}\right)}^{2}.$$

This formulation enables SURF to detect stable blob-like structures under scale and rotation changes. As a descriptor, SURF encodes local neighborhoods using Haar wavelet responses, yielding compact representations of gradient information. In deepfake detection, SURF is effective in capturing structural irregularities and unnatural textures introduced during face synthesis and blending.

Corner-Based Detectors (Harris, Shi-Tomasi, FAST) Corner points correspond to locations with significant intensity variation in multiple directions and are highly informative for forensic analysis

The Harris corner detector^48,49^ analyzes the second-moment matrix

3 $$\:M=\left[\begin{array}{cc}\sum\:\:{I}_{x}^{2}&\:\sum\:\:{I}_{x}{I}_{y}\\\:\sum\:\:{I}_{x}{I}_{y}&\:\sum\:\:{I}_{y}^{2}\end{array}\right]$$

and computes the response

4 $$\:R=\mathrm{d}\mathrm{e}\mathrm{t}\left(M\right)-k\left[\mathrm{t}\mathrm{r}\mathrm{a}\mathrm{c}\mathrm{e}\right(M){]}^{2},$$

where $\:k$ is a sensitivity parameter. Large positive values of $\:R$ indicate strong corners.

The Shi-Tomasi detector^50,51^ refines this approach by selecting corners based on the minimum eigenvalue of $\:M$, improving stability and repeatability.

FAST^13,52^ is a high-speed detector that examines a circle of 16 pixels around a candidate point. A point is classified as a corner if a contiguous set of pixels is significantly brighter or darker than the center pixel by a predefined threshold. FAST offers excellent efficiency while maintaining reliable corner localization.

These corner-based detectors precisely localize salient facial regions that are particularly sensitive to manipulation artifacts, aiding discrimination between real and fake images.

BRISK (Binary Robust Invariant Scalable Keypoints)BRISK^14,53^ is an efficient method for detecting and describing local features, well suited for real-time applications. Keypoints are detected using a FAST-based strategy, while the descriptor is formed through binary intensity comparisons over a predefined circular sampling pattern. For two sampling points$\:{p}_{i}$ and $\:{p}_{j}$, the binary test is defined as

5 $$\:{b}_{ij}=\left\{\begin{array}{ll}1,&\:I\left({p}_{i}\right)>I\left({p}_{j}\right),\\\:0,&\mathrm{otherwise},\end{array}\right.$$

where $\:I\left(p\right)$ denotes the pixel intensity at location $\:p$. The resulting binary string compactly encodes local texture and contrast information. BRISK provides robustness to scale and rotation changes, high computational efficiency, and resilience to common image distortions, making it effective for uncovering subtle manipulation traces.

The integration of SURF, corner-based detectors (FAST, Harris, and Shi–Tomasi), and BRISK forms a comprehensive local feature extraction strategy within the proposed framework. Each detector captures distinct aspects of local image structure—blob-like responses, corner geometry, and binary texture patterns—thereby increasing feature diversity and robustness. This complementary design enables the detection of a wide range of local facial characteristics and structural cues that may reveal manipulation artifacts introduced during deepfake generation.

#### Strong keypoint selection

To further enhance reliability and reduce redundancy, a keypoint strength selection strategy is applied. Specifically, only the strongest 20% of detected keypoints from each detector are retained based on their response magnitudes^44^. This selection process focuses the feature extraction on the most salient and discriminative regions while suppressing weak or noisy responses that may negatively impact representation quality. The retained keypoints from all detectors are subsequently aggregated to form a unified set of salient points for each image.

This multi-detector and strength-aware design strengthens the handcrafted feature representation and provides a robust foundation for the subsequent HOG-based Bag-of-Visual-Words construction and deep feature fusion, as described in the following sections.

#### Histogram of oriented gradients (HOG) descriptor computation

The HOG descriptor^11,44,45^ is employed to capture fine-grained local texture and structural information in facial images. HOG models the distribution of edge orientations, which is particularly sensitive to subtle inconsistencies introduced during facial manipulation and synthesis.

Given an image $\:I(x,y)$, the horizontal and vertical gradients are computed as

6 $$\:{G}_{x}=I(x+1,y)-I(x-1,y),\:{G}_{y}=I(x,y+1)-I(x,y-1).$$

The gradient magnitude and orientation are then obtained by

7 $$\:M(x,y)=\sqrt{{G}_{x}^{2}+{G}_{y}^{2}},\:\theta\:(x,y)=\mathrm{a}\mathrm{r}\mathrm{c}\mathrm{t}\mathrm{a}\mathrm{n}\left(\frac{{G}_{y}}{{G}_{x}}\right).$$

The image is divided into small spatial regions, referred to as cells, within which histograms of gradient orientations are accumulated and weighted by the corresponding gradient magnitudes. To improve robustness against illumination and contrast variations, neighboring cells are grouped into blocks, and histogram normalization is applied:

8 $$\:{h}_{\mathrm{n}\mathrm{o}\mathrm{r}\mathrm{m}}=\frac{h}{\sqrt{\Vert\:h{\Vert\:}_{2}^{2}+{\epsilon\:}^{2}}},$$

where $\:h$ denotes the unnormalized histogram vector and $\:\epsilon\:$ is a small constant for numerical stability.

HOG descriptors effectively encode edge structures, local shape information, and texture irregularities while remaining relatively invariant to global illumination changes. These characteristics make HOG particularly suitable for deepfake face detection, as synthetic manipulations often introduce subtle distortions in facial contours, edges, and gradient distributions.

In the proposed framework, HOG descriptors serve as a handcrafted forensic component and are integrated with the BoVW representation and deep CNN features, providing complementary low-level cues that enhance robustness and generalization in deepfake detection.

#### Visual vocabulary construction

To construct the BoVW representation, the proposed framework employs the K-means + + clustering algorithm^15^, an enhanced variant of the conventional K-means method designed to improve clustering stability and convergence. Unlike standard K-means, which initializes cluster centroids randomly and is therefore sensitive to initialization, K-means + + selects initial centroids that are well separated in the feature space. This strategy reduces the likelihood of poor local minima and leads to more consistent and discriminative clustering outcomes.

In this work, the HOG descriptors extracted around the selected local interest points are clustered using K-means + + to learn a visual vocabulary. The number of clusters is fixed at K = 500, where each cluster centroid represents a visual word corresponding to a frequently occurring local texture or structural pattern in facial images. Collectively, these centroids form a compact vocabulary that summarizes dominant handcrafted forensic features present in the training data.

Once the visual vocabulary is established, each image is encoded as a histogram of visual word occurrences by assigning its local HOG descriptors to the nearest cluster centroids. This histogram-based encoding constitutes the BoVW representation, providing a compact yet discriminative description of local image characteristics. Such representations enable effective comparison between real and deepfake images based on differences in their underlying local structural distributions and serve as the handcrafted feature component for subsequent fusion with deep CNN-based representations, as discussed in the following subsection.

#### BoVW image representation

After constructing the visual vocabulary, each facial image is represented using a BoVW encoding that summarizes the distribution of local patterns within the image )^8,9^. This representation is achieved through histogram creation and normalization.

First, each HOG descriptor extracted from an image is assigned to its nearest visual word by measuring the Euclidean distance to the vocabulary centroids. Let $\:\mathcal{V}=\left\{{\mu\:}_{1},{\mu\:}_{2},\dots\:,{\mu\:}_{K}\right\}$ denote the visual vocabulary learned via $\:K$-means++, where $\:K=500$. Each local descriptor $\:{\mathrm{h}}_{ik}$ is mapped to the closest centroid $\:{\mu\:}_{j}$, thereby associating it with a specific visual word.

Next, a histogram of visual word occurrences is constructed for each image:

9 $$\:{\mathrm{f}}_{\mathrm{B}\mathrm{o}\mathrm{V}\mathrm{w}}\left({I}_{i}\right)=\left[{c}_{1},{c}_{2},\dots\:,{c}_{K}\right]$$

where $\:{c}_{j}$ denotes the frequency of descriptors assigned to the $\:j$-th visual word. This histogram captures the distribution of dominant local textures and structural patterns within the image, which are indicative of potential manipulation artifacts.

To ensure comparability across images with different numbers of detected keypoints, the histogram is normalized, typically using $\:{L}_{1}$ or $\:{L}_{2}$ normalization:

10 $$\:{\stackrel{\prime }{\mathrm{f}}}_{\mathrm{B}\mathrm{o}\mathrm{V}\mathrm{W}}\left({I}_{i}\right)=\frac{{\mathrm{f}}_{\mathrm{B}\mathrm{o}\mathrm{V}\mathrm{W}}\left({I}_{i}\right)}{\lVert{\mathrm{f}}_{\mathrm{B}\mathrm{o}\mathrm{V}\mathrm{W}}\left({I}_{i}\right)\lVert}$$

Normalization mitigates the influence of image size and keypoint density, resulting in a stable and scale invariant handcrafted feature representation.

The normalized BoVW feature vector serves as the handcrafted forensic descriptor in the proposed framework and is subsequently fused with deep CNN-based features to form a comprehensive hybrid representation for deepfake face detection.

### Deep feature extraction and fusion (Multi-CNN Module)

**Fig. 3:**
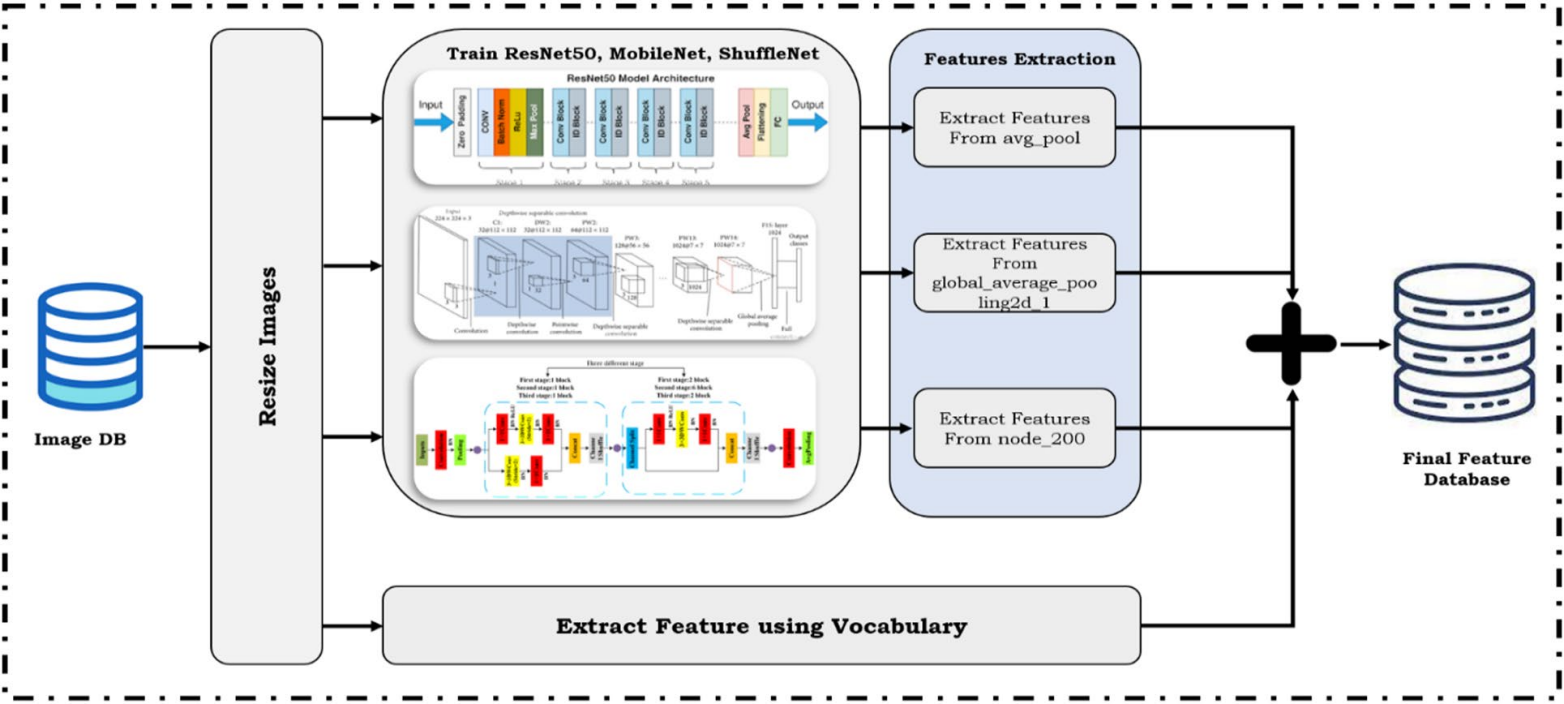
Overview of the proposed multi-CNN deep feature extraction and feature-level fusion process.

The second stage of the proposed framework focuses on extracting deep semantic representations from facial images using multiple pre-trained Convolutional Neural Network (CNN) architectures and fusing them to capture complementary discriminative information. This multi-CNN strategy is designed to leverage the strengths of different network architectures while maintaining computational efficiency. The overall workflow of the deep feature extraction and fusion module is illustrated in Fig. 3.

As shown in Fig. 3, facial images from the database are first resized to meet the input requirements of each CNN model. Three widely adopted pre-trained architectures—ResNet-50, MobileNet, and ShuffleNet—are then fine-tuned using transfer learning on the target dataset. These models were selected due to their complementary design characteristics, balancing representational depth, efficiency, and robustness to diverse facial variations.

#### Pre-trained CNN models

To extract robust and complementary deep representations for deepfake face detection, this work employs three widely adopted pre-trained Convolutional Neural Network (CNN) architectures: ResNet-50, MobileNet, and ShuffleNet. These models are selected due to their complementary design philosophies, collectively balancing representational capacity, computational efficiency, and robustness.

ResNet-50^16^ is a deep CNN architecture consisting of 50 layers and is distinguished by the use of residual connections, which facilitate efficient gradient propagation during training. Instead of learning a direct mapping $\:H\left(x\right)$, residual learning reformulates the objective as

11 $$\:H\left(x\right)=F\left(x\right)+x$$

where $\:F\left(x\right)$ denotes the residual function to be learned. This formulation effectively mitigates the degradation problem observed in very deep networks, allowing ResNet-50 to capture high-level semantic facial features that are particularly useful for detecting manipulation artifacts.

MobileNet^17^ is a lightweight CNN architecture designed for efficiency in vision-based tasks, especially in scenarios requiring fast inference and low computational overhead. MobileNet employs depthwise separable convolutions, which decompose a standard convolution into a depthwise convolution followed by a pointwise convolution:

12 $${\mathrm{Conv}}_{\mathrm{std}}\approx{\mathrm{Conv}}_{\mathrm{depthwise}}+{\mathrm{Conv}}_{\mathrm{pointwise:}}$$

This decomposition significantly reduces parameter count and floating-point operations while maintaining competitive accuracy, making MobileNet well suited for resource-constrained and real-time deepfake detection applications.

ShuffleNet^18^ is another efficient CNN architecture optimized for mobile and edge deployment. It introduces pointwise group convolutions to reduce computational complexity, combined with a *channel shuffling* operation that enables effective information exchange across channel groups. This design achieves a favorable trade-off between speed and accuracy, allowing ShuffleNet to extract discriminative yet low-cost visual features.

#### Transfer learning strategy

To effectively adapt deep representations to the deepfake face detection task while mitigating overfitting, all CNN models are initialized with ImageNet pre-trained weights and fine-tuned using transfer learning. Fine-tuning allows the networks to retain generic visual knowledge while learning task-specific facial manipulation patterns from the target dataset.

The training configuration is selected to ensure stable convergence and computational efficiency. The initial learning rate is set to 0.001, the mini-batch size is fixed at 20, and the maximum number of epochs is limited to 10. These settings balance learning stability and training speed while preventing excessive parameter updates. In addition, early stopping is employed to halt training when validation performance no longer improves, further reducing the risk of overfitting.

To enhance generalization, data augmentation techniques^32^ are applied during training, including horizontal flipping, slight rotations, and contrast adjustments. These augmentations increase data diversity and improve robustness to variations in pose, illumination, and facial appearance commonly observed in real-world scenarios. Through this transfer learning strategy, each CNN model learns discriminative deep features tailored to the deepfake detection problem while maintaining robustness across diverse conditions.

#### Feature-level fusion

To exploit the complementary strengths of different CNN architectures, the proposed framework adopts a feature-level fusion strategy. Deep features are extracted from selected high-level layers of each CNN model—ResNet-50, MobileNet, and ShuffleNet—specifically from layers that capture rich semantic and spatial information. These layers are chosen to balance discriminative power and robustness while avoiding redundancy from low-level representations.

Let ${\mathrm{f}}_{R},{\mathrm{f}}_{M}$, and ${\mathrm{f}}_{S}$ denote the feature vectors extracted from ResNet-50, MobileNet, and ShuffleNet, respectively. The fused deep feature representation is constructed via concatenation:

13 $${\mathrm{F}}_{\mathrm{Deep}}=\left[{\mathrm{f}}_{R}\lVert{\mathrm{f}}_{M}\lVert{\mathrm{f}}_{S}\right]$$

This concatenation preserves complementary information from each architecture, enabling the model to capture diverse facial characteristics ranging from deep semantic inconsistencies to lightweight structural cues.

Feature-level fusion is preferred over decision-level fusion because it retains richer discriminative information prior to classification. While decision-level fusion combines only final predictions, feature-level fusion allows the classifier to learn optimal decision boundaries directly from the combined feature space. More importantly, this strategy facilitates seamless integration with handcrafted forensic features, enabling deep semantic representations to complement low-level texture and structural artifacts extracted by the BoVW-HOG module.

### Hybrid feature integration

To form a comprehensive representation, the fused deep feature vector is combined with the handcrafted BoVW-HOG feature vector through feature concatenation:

14 $${\mathrm{F}}_{\mathrm{Hybrid}}=\left[{\mathrm{F}}_{\mathrm{B}\mathrm{o}\mathrm{V}\mathrm{W}}\Vert\:{\mathrm{F}}_{\mathrm{Deep}}\right]$$

This hybrid feature integration unifies low-level forensic cues-such as local texture irregularities and gradient inconsistencies-with high-level semantic patterns learned by deep CNNs^54^. The resulting feature vector captures a broader spectrum of manipulation artifacts than either representation alone.

Hybridization improves generalization by reducing reliance on manipulation-specific deep patterns and incorporating stable handcrafted descriptors that are less sensitive to dataset bias. As a result, the framework remains robust against unseen or evolving deepfake generation techniques while maintaining computational efficiency and interpretability.

### Classification using support vector machine (SVM)

To discriminate between real and deepfake facial images, a SVM classifier is employed on the fused hybrid feature representation. Several kernel functions are evaluated to effectively model the underlying data distribution and decision boundaries^19^. Specifically, a linear kernel is considered for approximately linearly separable feature spaces, while quadratic and cubic polynomial kernels are used to capture moderate nonlinear relationships^55,56^. In addition, the Gaussian Radial Basis Function (RBF) kernel is explored to handle highly nonlinear feature interactions arising from the fusion of deep and handcrafted descriptors. This kernel diversity allows the classifier to adapt to varying levels of feature-space complexity.

To identify the optimal SVM configuration, a systematic grid search is conducted over kernel types and key regularization parameters. Model selection is guided by validation-based performance metrics, which provide a balanced evaluation under class imbalance and across different decision thresholds. Compared with end-to-end deep learning classifiers, the SVM-based classification strategy offers stable optimization behavior, a reduced risk of overfitting, and enhanced interpretability^56,57^. By decoupling feature extraction from classification, the proposed framework leverages the representational power of deep features while preserving robust, transparent, and reliable decision-making, making it particularly suitable for forensic and security-critical deepfake detection applications.

Algorithm 1 summarizes the complete workflow of the proposed hybrid deepfake face detection framework, integrating handcrafted BoVW–HOG forensic descriptors with fused multi-CNN deep representations. The algorithm details the sequential stages of feature extraction, feature-level fusion, and SVM-based classification used to achieve robust, interpretable, and generalizable deepfake detection.

**Algorithm 1:**
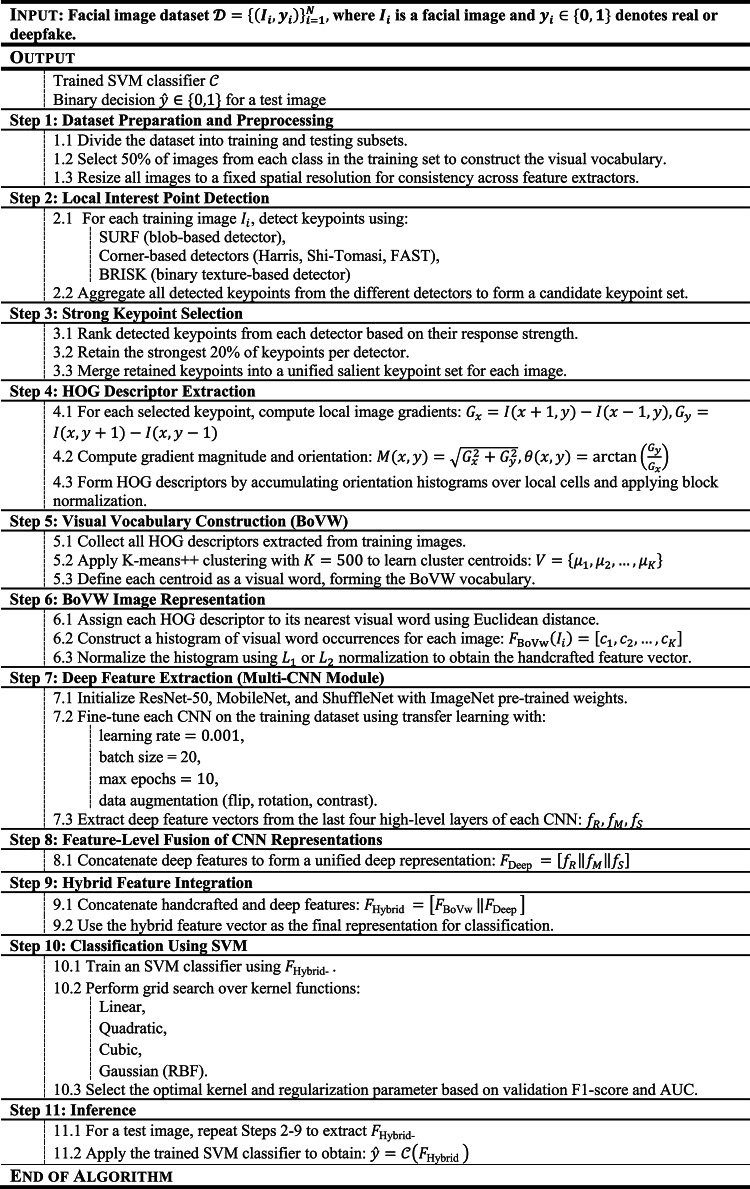
Hybrid BoVW–HOG and multi-CNN feature fusionframework for deepfake face detection

## Experimental results and discussion

### Datasets description

The experimental evaluation was performed on six publicly available face manipulation datasets, organized according to dataset scale and classification type, as summarized in Table 2. This structured organization enables a systematic assessment of the proposed model under varying data volumes, manipulation characteristics, and levels of visual difficulty.

Based on the total number of images, the datasets are grouped into big-scale (≥ 100 K samples), medium-scale (10–100 K samples), and small-scale (< 10 K samples) categories. This explicit size-based clustering facilitates evaluation of both scalability under data-rich conditions and robustness in data-scarce scenarios.

The 130 K Real vs. Fake Face^20^ and Real vs. AI-Generated Faces^21^ datasets fall into the big-scale category and serve as large-scale benchmarks for real–fake and AI-generated face detection. These datasets exhibit high intra-class variability and are primarily used to assess the model’s stability and generalization on diverse data distributions. The Real/Fake Cropped Faces^22^ and RVF10K^23^ datasets constitute the medium-scale category, where RVF10K provides a balanced baseline benchmark, while the cropped faces dataset emphasizes fine-grained artifact detection under reduced contextual information.

The Human Faces^25^ and Fake vs. Real Faces (Hard)^24^ datasets are categorized as small-scale benchmarks. The former focuses on AI-generated face detection, whereas the latter contains visually ambiguous and low-quality samples, posing increased detection difficulty. These datasets are particularly suitable for evaluating robustness under limited training data and heightened visual uncertainty.

In addition to scale-based grouping, the datasets are further categorized by classification type, including standard real–fake detection, AI-generated face detection, cropped face analysis, and hard/challenging cases. This dual clustering strategy ensures comprehensive evaluation across complementary detection scenarios rather than reliance on a single benchmark. For all datasets, a consistent 70:30 train–test split is adopted, and class-wise distributions are reported to ensure transparency and reproducibility.

**Table 2:** Summary of datasets used in this study, grouped by scale and classification type.

Dataset Name	Size Category	Classification Type	Class	Total	Train	Test
130 K Real vs. Fake Face^20^	Big-scale	Binary (Real vs. Fake)	Fake	63,569	44,498	19,071
			Real	70,000	49,000	21,000
Real vs. AI-Generated Faces^21^	Big-scale	Binary (Real vs. AI-Generated)	Fake	50,954	35,668	15,286
			Real	70,000	49,000	21,000
Real/Fake Cropped Faces^22^	Medium-scale	Binary (Real vs. Fake, Cropped)	Fake	33,838	23,687	10,151
			Real	34,641	24,249	10,392
RVF10K^23^ (Real vs. Fake Faces – 10k)	Medium-scale	Binary (Real vs. Fake)	Fake	5,000	3,500	1,500
			Real	5,000	3,500	1,500
Human Faces^25^	Small-scale	Binary (Real vs. AI-Generated)	AI-Generated	4,630	3,241	1,389
			Real	5,000	3,500	1,500
Fake vs. Real Faces (Hard)^24^	Small-scale	Binary (Hard / Challenging Cases)	Fake	700	490	210
			Real	589	412	177

### Performance metrics categorization

To ensure a comprehensive and transparent evaluation, the performance measures used in this study are organized into three complementary categories: dataset-level metrics, class-wise metrics, and visual diagnostic tools. This categorization enables a clear assessment of overall accuracy, class balance, and discriminative capability across datasets of varying scale and difficulty.

#### Dataset-level performance metrics

Overall classification effectiveness is assessed using overall accuracy and weighted accuracy, which measure the proportion of correctly classified samples while accounting for class distribution. Macro-averaged precision, recall, and F1-score are employed to evaluate balanced performance across classes, whereas micro F1-score reflects global prediction consistency^58,59^. To assess robustness under class imbalance, Balanced Error Rate (BER) and G-Mean are reported. In addition, Global Kappa (Cohen’s Kappa) is used to quantify agreement beyond chance, and 95% confidence intervals (CI) are provided to ensure statistical reliability and reproducibility of the results.

#### Class-wise performance metrics

To analyze model behavior at the class level, accuracy, precision, recall (sensitivity), and specificity are computed separately for each class. The F1-score is used to summarize the trade-off between precision and recall, while the area under the ROC curve (AUC) evaluates class separability independent of decision thresholds^60,61^. Additional robustness indicators include error rate, balanced accuracy, Global Kappa, G-Mean, and Youden’s J statistic, which collectively provide deeper insight into class discrimination and misclassification patterns^62^.

#### Visual and threshold-independent evaluation

To complement numerical metrics, visual diagnostic tools are employed. Confusion matrices (CM) illustrate correct and incorrect predictions for each class, enabling intuitive inspection of misclassification trends. Receiver Operating Characteristic (ROC) curves assess discriminative capability across varying thresholds, while Precision–Recall (PR) curves emphasize performance under class imbalance and challenging detection scenarios^55,61,63,64^. These visual analyses support interpretability and reinforce the quantitative findings.

All experiments were carried out on a Lenovo Legion Pro 5 workstation equipped with a 14th Gen Intel Core i7-14650HX CPU, an NVIDIA RTX 4060 GPU (8 GB), 32 GB DDR5 RAM, and a 1 TB NVMe SSD, running Windows 11 Pro. All implementations were performed in MATLAB R2024b, with GPU acceleration enabled.

### Detailed performance analysis on small-scale datasets

The Human Faces and Fake vs. Real Faces (Hard) datasets represent the most challenging evaluation scenarios due to limited training data, visual ambiguity, and high intra-class similarity. Performance on these datasets therefore provides a strong indicator of the proposed model’s robustness, generalization capability, and resistance to overfitting under data-scarce and adversarial conditions.

#### Quantitative performance evaluation on human faces dataset

The Human Faces dataset targets AI-generated face detection in a small-scale setting. As reported in Table 3, the proposed model achieves an overall accuracy of 99.10%, with identical weighted and micro F1-scores of 0.9910, indicating highly consistent predictions across both classes. The macro precision (0.9912) and macro recall (0.9908) are closely aligned, confirming balanced classification performance without bias toward either real or AI-generated images.

The Global Kappa value of 0.9820 demonstrates excellent agreement beyond chance, while the extremely low Balanced Error Rate (BER) of 0.0092 highlights minimal misclassification despite the limited dataset size. In addition, the G-Mean of 0.9908 confirms stable class-wise sensitivity. The narrow 95% confidence interval [0.9868, 0.9939] further indicates strong statistical reliability and robustness of the reported results.

A deeper class-wise analysis (Table 4) shows that AI-generated images achieve a precision of 0.99564 and recall of 0.98560, resulting in a high F1-score of 0.99059, while real images achieve a recall of 0.99600 and F1-score of 0.99137. The identical AUC value of 0.99080 for both classes indicates excellent separability between real and AI-generated samples. Furthermore, the high Youden’s J statistic (0.98160) confirms strong diagnostic effectiveness. Collectively, these results demonstrate that the proposed model maintains high discriminative power and balanced performance even under data-scarce conditions involving modern generative models.

#### Quantitative performance evaluation on fake vs. real faces (hard) dataset

The Fake vs. Real Faces (Hard) dataset is explicitly designed to challenge detection models through low-quality samples, adversarial artifacts, and visually ambiguous manipulations. As shown in Table 3, the proposed model achieves an overall accuracy of 97.67%, which is notable given the increased difficulty of this benchmark. The macro precision (0.9773), macro recall (0.9759), and macro F1-score (0.9765) remain closely aligned, indicating balanced performance across real and fake classes.

The Global Kappa of 0.9531 confirms strong agreement beyond chance, while the BER of 0.0241 reflects the expected but controlled increase in misclassification relative to less challenging datasets. The G-Mean of 0.9759 indicates that class-wise sensitivity remains stable despite visual degradation. The reported 95% confidence interval [0.9564, 0.9877] further confirms the statistical reliability of the achieved performance.

From a class-wise perspective (Table 4), fake images achieve a recall of 0.98571, whereas real images achieve a slightly lower recall of 0.96610, highlighting the asymmetric difficulty inherent in this dataset. Nevertheless, both classes maintain high AUC values of 0.97591, confirming strong discriminative capability even under adverse and visually ambiguous conditions. The Youden’s J statistic (0.95182) further demonstrates effective separation between real and fake classes despite reduced visual cues.

#### Confusion matrix analysis on small-scale datasets

**Fig. 4:**
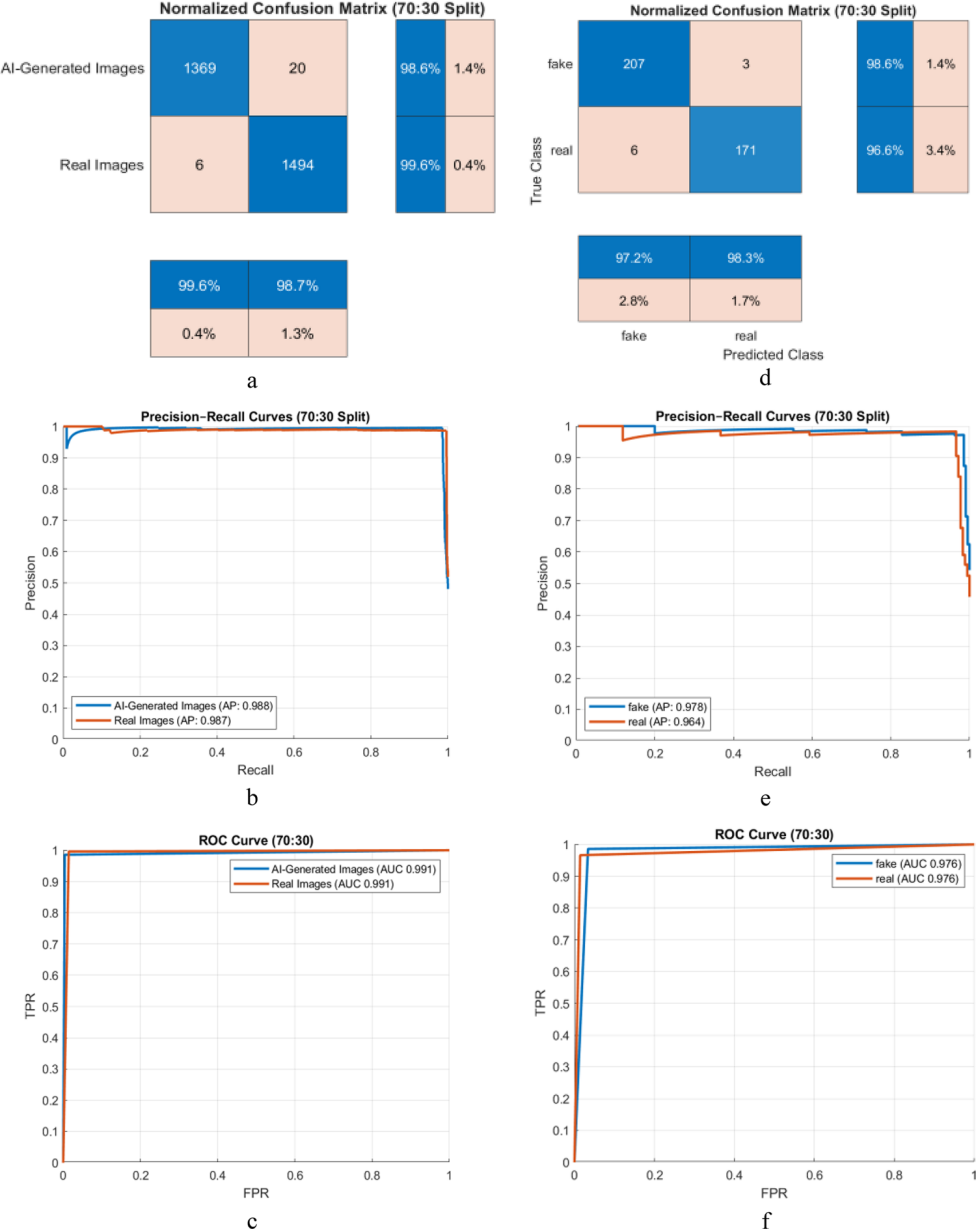
(**a**–**c**) Normalized confusion matrices, precision–recall curves, and ROC curves for the Human Faces dataset, and (d, e, f) corresponding results for the Fake vs. Real Faces (Hard) dataset, using a 70:30 train–test split. Both represent small-scale benchmark evaluations

The normalized confusion matrices shown in Fig. 4a and d provide detailed insight into the class-level prediction behavior of the proposed model on small-scale benchmarks. For the Human Faces dataset (Fig. 4a), near-perfect classification performance is observed. AI-generated images are correctly identified with a recall of approximately 98.6%, while real images achieve an even higher recall of 99.6%, resulting in extremely low misclassification rates. The strong diagonal dominance reflects excellent separability between real and AI-generated samples and is consistent with the high Global Kappa (0.9820) and low BER (0.0092) values reported in Table 3.

In contrast, the Fake vs. Real Faces (Hard) dataset (Fig. 4d) presents a more challenging detection scenario due to visually ambiguous and low-quality samples. Fake images are correctly classified with a recall of approximately 98.6%, whereas real images achieve a slightly lower recall of 96.6%. This mild asymmetry—where real images are more prone to misclassification—is expected, as genuine samples often lack distinctive manipulation artifacts. Nevertheless, the clear diagonal dominance and balanced prediction rates confirm that the proposed framework effectively captures robust forensic features even under adverse conditions.

.

**Table 3:** Overall performance comparison on small-scale datasets.

Dataset	Overall Accuracy	Weighted accuracy	Macro precision	Macro recall	Macro F1	Weighted F1	Micro F1	Global Kappa	BER	G-Mean	95% Confidence Interval
Human Faces	99.10%	99.10%	0.9912	0.9908	0.9910	0.9910	0.9910	0.9820	0.0092	0.9908	[0.9868, 0.9939]
Fake vs. Real Faces (Hard)	97.67%	97.67%	0.9773	0.9759	0.9765	0.9767	0.9767	0.9531	0.0241	0.9759	[0.9564, 0.9877]

**Table 4:** Class-wise performance metrics on small-scale datasets.

Dataset	Class	Accuracy	Precision	Recall	Specificity	F1-score	AUC	Error Rate	Balanced Acc	Kappa	G-Mean	Youden’s J
Human Faces	AI-Generated Images	0.98560	0.99564	0.98560	0.99600	0.99059	0.99080	0.014399	0.99080	0.98204	0.99079	0.98160
	Real Images	0.99600	0.98679	0.99600	0.98560	0.99137	0.99080	0.00400	0.99080	0.98204	0.99079	0.98160
	Average	0.99080	0.99121	0.99080	0.99080	0.99098	0.99080	0.009199	0.99080	0.98204	0.99079	0.98160
Fake vs. Real Faces (Hard)	Fake	0.98571	0.97183	0.98571	0.96610	0.97872	0.97591	0.014286	0.97591	0.95396	0.97586	0.95182
	Real	0.96610	0.98276	0.96610	0.98571	0.97436	0.97591	0.033898	0.97591	0.95396	0.97586	0.95182
	Average	0.97591	0.97729	0.97591	0.97591	0.97654	0.97591	0.024092	0.97591	0.95396	0.97586	0.95182

.

#### Precision–recall and ROC curve analysis on small-scale datasets

The precision–recall (PR) curves shown in Fig. 4b for the Human Faces dataset demonstrate consistently high precision across nearly the entire recall range for both classes. The average precision (AP) reaches approximately 0.988 for AI-generated images and 0.987 for real images, indicating a strong precision–recall balance even as recall approaches unity. The minor precision degradation observed only at very high recall values reflects the expected trade-off near the decision boundary and does not materially affect overall performance.

The corresponding ROC curves in Fig. 4c exhibit near-ideal behavior, with both classes achieving an AUC of approximately 0.991. The curves rise sharply toward the top-left corner, confirming high true positive rates at very low false positive rates and reinforcing the strong class separability observed in the confusion matrix.

For the Fake vs. Real Faces (Hard) dataset, the PR curves in Fig. 4e show slightly increased variability, reflecting the higher visual uncertainty inherent in this benchmark. Despite this challenge, the average precision remains high, reaching approximately 0.978 for fake images and 0.964 for real images. The corresponding ROC curves in Fig. 4f1 achieve an AUC of approximately 0.976, confirming strong discriminative capability even under adversarial and degraded conditions. Although marginally less steep than those of the Human Faces dataset, the curves still demonstrate high true positive rates at low false positive rates.

#### Comparative interpretation on small-scale datasets

A comparative analysis across both small-scale datasets reveals that while the Human Faces dataset benefits from clearer generative artifacts and structured synthesis patterns, the Hard dataset introduces substantial ambiguity through degraded image quality and adversarial manipulations. Despite these challenges, the proposed model consistently achieves high accuracy (> 97%), strong agreement metrics, and stable G-Mean values, demonstrating robust generalization under limited data availability.

Importantly, the modest performance gap between the two datasets indicates that the proposed framework does not rely solely on dataset scale or visual clarity, but instead learns discriminative representations that generalize across diverse and challenging real–fake detection scenarios.

### Detailed performance analysis on medium-scale datasets

The Real/Fake Cropped Faces and Real vs. Fake Faces–10k (RVF10K) datasets represent medium-scale evaluation scenarios, designed to assess the proposed model under moderate data availability and varying degrees of visual context. These datasets are particularly important for evaluating robustness to partial facial information and balanced benchmark conditions, thereby bridging the gap between large-scale datasets and more challenging small-scale settings.

#### Quantitative performance evaluation on real/fake cropped faces dataset

The Real/Fake Cropped Faces dataset emphasizes region-level facial analysis, where full facial context is partially removed. As reported in Table 5, the proposed model achieves an overall accuracy of 97.42%, with identical weighted and micro F1-scores of 0.9742, indicating stable and consistent predictions across classes. The macro precision (0.9744) and macro recall (0.9743) are nearly identical, confirming balanced classification behavior without class bias.

The Global Kappa value of 0.9484 indicates strong agreement beyond chance, while the Balanced Error Rate (BER) of 0.0257 reflects a modest but expected increase in misclassification due to reduced facial context. The G-Mean of 0.9743 confirms stable class-wise sensitivity, and the narrow 95% confidence interval [0.9719, 0.9763] demonstrates statistical reliability.

Class-wise analysis (Table 6) shows that fake images achieve a recall of 0.98641, whereas real images achieve a recall of 0.96228, indicating mild asymmetry in class difficulty. Nevertheless, both classes maintain identical AUC values of 0.97434, and the Youden’s J statistic (0.94868) confirms strong diagnostic effectiveness under region-focused conditions.

#### Quantitative performance evaluation on real vs. fake faces–10k (RVF10K) Dataset

The RVF10K dataset represents a widely used balanced medium-scale benchmark for standard real–fake face detection. As shown in Table 5, the proposed model achieves an overall accuracy of 98.47%, demonstrating strong generalization under moderate data availability. The macro precision (0.9850), macro recall (0.9847), and macro F1-score (0.9847) are tightly aligned, indicating balanced performance across classes.

The Global Kappa value of 0.9693 reflects excellent agreement beyond chance, while the low BER of 0.0153 highlights minimal misclassification. The G-Mean of 0.9846 further confirms stable and symmetric sensitivity across real and fake classes. The 95% confidence interval [0.9796, 0.9885] underscores the robustness and reproducibility of the reported results.

From a class-wise perspective (Table 6), fake images achieve a precision of 0.99863 and recall of 0.97067, whereas real images achieve a recall of 0.99867 with slightly lower precision (0.97147). This symmetric behavior indicates that the model does not favor either class. The identical AUC value of 0.98467 and Youden’s J statistic (0.96933) confirm strong discriminative capability under standard detection conditions.

#### Confusion matrix analysis on medium-scale datasets

**Fig. 5:**
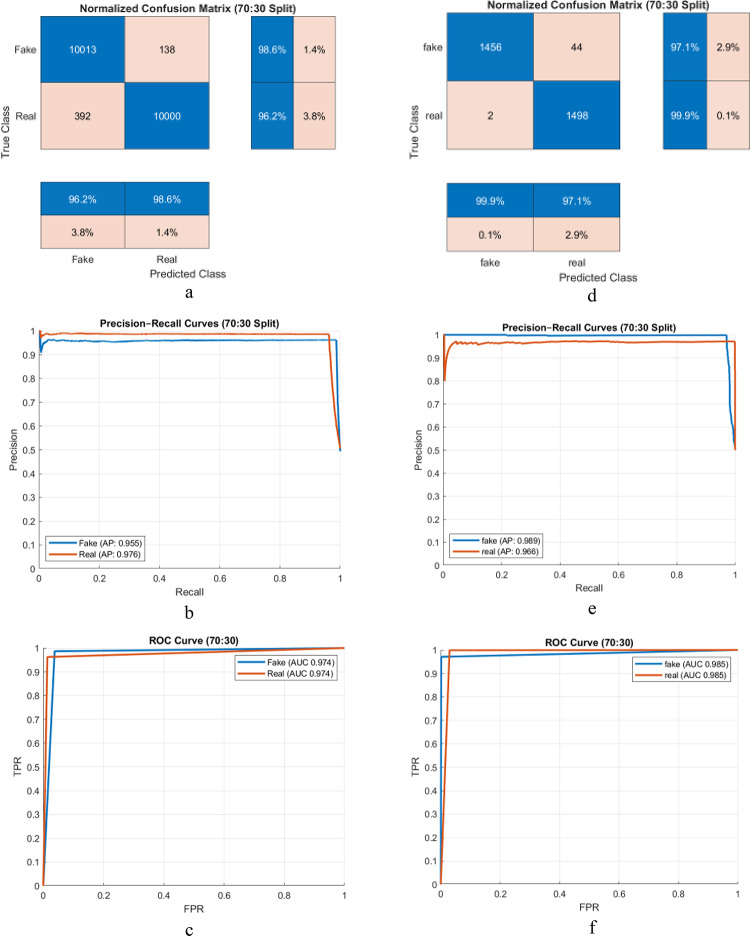
(**a**–**c**) Normalized confusion matrices, precision–recall curves, and ROC curves for the Real/Fake Cropped Faces dataset, and (**d**–**f**) corresponding results for the Real vs. Fake Faces – 10k (RVF10K) dataset, using a 70:30 train–test split. Both represent medium-scale benchmark evaluations

The normalized confusion matrices shown in Fig. 5a,d illustrate class-level prediction behavior on the medium-scale benchmarks. For the Real/Fake Cropped Faces dataset (Fig. 5a), fake images are correctly classified with a recall of approximately 98.6%, while real images achieve a recall of approximately 96.2%. The slightly higher misclassification rate for real samples can be attributed to the reduced availability of global facial context when only localized regions are analyzed. Despite this challenge, the strong diagonal dominance indicates reliable class separation, consistent with the maintained AUC (≈ 0.974) and G-Mean (≈ 0.974) values.

**Table 5:** Overall performance comparison on medium-scale datasets.

Dataset	Overall Accuracy	Weighted Accuracy	Macro Precision	Macro Recall	Macro F1	Weighted F1	Micro F1	Global Kappa	BER	G-Mean	95% Confidence Interval
Real/Fake Cropped Faces	97.42%	97.42%	0.9744	0.9743	0.9742	0.9742	0.9742	0.9484	0.0257	0.9743	[0.9719, 0.9763]
Real vs. Fake Faces – 10k (RVF10K)	98.47%	98.47%	0.9850	0.9847	0.9847	0.9847	0.9847	0.9693	0.0153	0.9846	[0.9796, 0.9885]

**Table 6:** Class-wise performance metrics on medium-scale datasets.

Dataset	Class	Accuracy	Precision	Recall	Specificity	F1-score	AUC	Error Rate	Balanced Acc	Kappa	G-Mean	Youden’s J
Real/Fake Cropped Faces	Fake	0.98641	0.96233	0.98641	0.96228	0.97422	0.97434	0.013595	0.97434	0.94841	0.97427	0.94868
	Real	0.96228	0.98639	0.96228	0.98641	0.97418	0.97434	0.037721	0.97434	0.94841	0.97427	0.94868
	Average	0.97434	0.97436	0.97434	0.97434	0.97420	0.97434	0.025658	0.97434	0.94841	0.97427	0.94868
Real vs. Fake Faces – 10k (RVF10K)	Fake	0.97067	0.99863	0.97067	0.99867	0.98445	0.98467	0.029333	0.98467	0.96936	0.98457	0.96933
	Real	0.99867	0.97147	0.99867	0.97067	0.98488	0.98467	0.001333	0.98467	0.96936	0.98457	0.96933
	Average	0.98467	0.98505	0.98467	0.98467	0.98466	0.98467	0.015333	0.98467	0.96936	0.98457	0.96933

In contrast, the confusion matrix for the RVF10K dataset shown in Fig. 5d demonstrates near-ideal classification behavior. Fake images achieve a recall of approximately 97.1%, while real images reach approximately 99.9%, resulting in extremely low misclassification for genuine samples. These results are in close agreement with the high overall accuracy, low BER, and strong Global Kappa values reported in Table 5, confirming the robustness of the proposed framework on balanced medium-scale data.

#### Precision–recall and roc curve analysis on medium-scale datasets

The precision–recall (PR) curves for the Real/Fake Cropped Faces dataset shown in Fig. 5b indicate strong precision across most of the recall range. The average precision (AP) reaches approximately 0.955 for fake images and 0.976 for real images. A moderate precision degradation is observed only at very high recall levels, which is expected due to ambiguity introduced by partial facial visibility. The corresponding ROC curves in Fig. 5c achieve an AUC of approximately 0.974 for both classes, confirming effective separability even when global facial cues are partially removed.

For the RVF10K dataset, the PR curves in Fig. 5e demonstrate consistently high precision across nearly the entire recall range, with AP values of approximately 0.989 for fake images and 0.966 for real images. The corresponding ROC curves in Fig. 5f exhibit near-ideal behavior, achieving an AUC of approximately 0.985 for both classes. The steep ascent toward the top-left region confirms high sensitivity at low false-positive rates, consistent with the strong quantitative performance metrics reported earlier.

#### Comparative interpretation on medium-scale datasets

Comparing both medium-scale datasets highlights the effect of visual context availability. While RVF10K benefits from complete facial information and achieves slightly higher accuracy and lower BER, the Real/Fake Cropped Faces dataset introduces increased uncertainty due to localized facial regions. Despite this added complexity, the proposed model consistently maintains accuracy above 97%, balanced macro metrics, and strong agreement measures across both datasets.

### Detailed performance analysis on big-scale datasets

The Real vs. AI-Generated Faces and 130 K Real vs. Fake Face datasets represent big-scale evaluation scenarios, each containing more than 100 K samples. These datasets are essential for evaluating the scalability, robustness, and stability of the proposed model under large data volumes, diverse facial characteristics, and realistic manipulation distributions. Performance on these datasets provides strong evidence of the model’s ability to generalize without overfitting and to maintain balanced behavior at scale.

#### Quantitative performance evaluation on real vs. AI-generated faces dataset

The Real vs. AI-Generated Faces dataset focuses on large-scale detection of synthetically generated faces, including GAN- and diffusion-based images. As reported in Table 7, the proposed model achieves an overall accuracy of 99.12%, with identical weighted and micro F1-scores of 0.9912, indicating highly consistent predictions across both classes. The macro precision (0.9909) and macro recall (0.9910) are tightly aligned, confirming balanced classification performance without bias toward either real or AI-generated samples.

The Global Kappa value of 0.9819 demonstrates excellent agreement beyond chance, while the extremely low Balanced Error Rate (BER) of 0.0090 highlights minimal misclassification despite the large dataset size and diversity. The G-Mean of 0.9910 further confirms stable sensitivity across both classes. The narrow 95% confidence interval [0.9901, 0.9921] indicates strong statistical reliability.

Class-wise analysis (Table 8) shows that fake images achieve a recall of 0.98999, while real images achieve a recall of 0.99200, demonstrating symmetric and reliable detection behavior. Both classes achieve an identical AUC of 0.99100, confirming excellent separability between real and AI-generated faces. The high Youden’s J statistic (0.98199) further highlights strong diagnostic effectiveness. These results confirm that the proposed model scales effectively to large datasets while maintaining high discriminative power.

#### Quantitative Performance evaluation on 130 K real vs. fake face dataset

The 130 K Real vs. Fake Face dataset represents a large-scale benchmark for standard real–fake face detection under diverse acquisition conditions and manipulation types. As shown in Table 7, the proposed model achieves an overall accuracy of 97.59%, reflecting strong generalization under realistic large-scale conditions. The macro precision (0.9756), macro recall (0.9765), and macro F1-score (0.9759) remain closely aligned, indicating balanced classification performance across classes.

The Global Kappa value of 0.9517 confirms strong agreement beyond chance, while the BER of 0.0235 reflects a controlled increase in misclassification compared to AI-generated datasets—an expected outcome given the higher diversity and subtlety of real-world manipulations. The G-Mean of 0.9764 confirms stable class-wise sensitivity, and the 95% confidence interval [0.9743, 0.9773] underscores statistical reliability.

From a class-wise perspective (Table 8), fake images achieve a recall of 0.98836, while real images achieve a slightly lower recall of 0.96457, indicating mild asymmetry in class difficulty. Nevertheless, both classes maintain identical AUC values of 0.97647, confirming strong discriminative capability across diverse facial appearances. The Youden’s J statistic (0.95293) further demonstrates effective separation under large-scale conditions.

#### Confusion matrix analysis on big-scale datasets

**Fig. 6:**
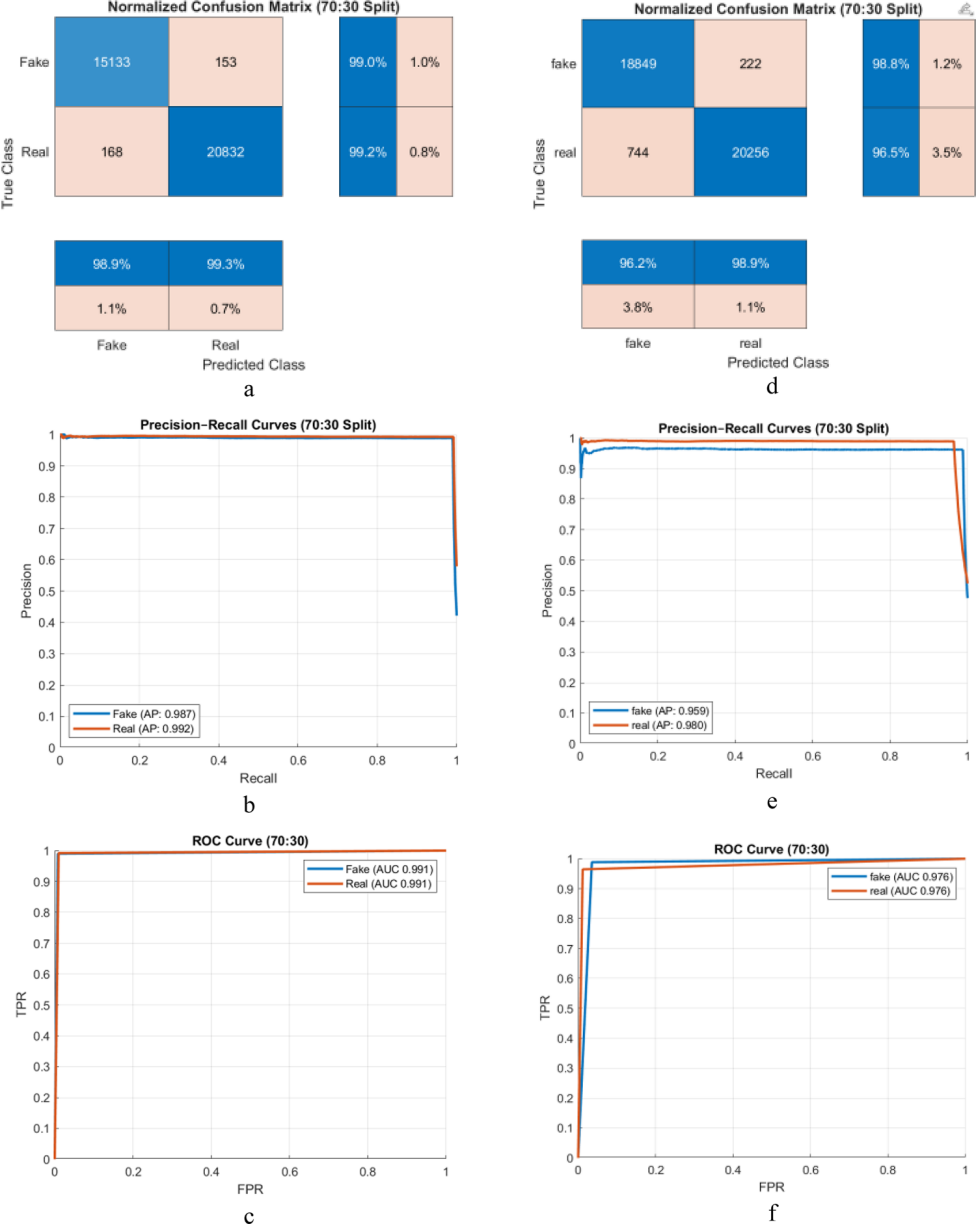
(**a**–**c**) Normalized confusion matrices, precision–recall curves, and ROC curves for the Real vs. AI-Generated Faces dataset, and (**d**–**f**) corresponding results for the 130 K Real vs. Fake Face dataset, using a 70:30 train–test split. Both represent big-scale benchmark evaluations

The normalized confusion matrices presented in Fig. 6a, d provide insight into class-level prediction behavior under large-scale evaluation conditions. For the Real vs. AI-Generated Faces dataset (Fig. 6a), AI-generated images are correctly classified with a recall of approximately 99.0%, while real images achieve a recall of approximately 99.2%, resulting in extremely low confusion between classes. The strong diagonal dominance and symmetric error distribution indicate excellent separability, consistent with the high AUC, strong Global Kappa, and low BER values reported in Tables 4 and 5.

In contrast, the 130 K Real vs. Fake Face dataset shown in Fig. 6d exhibits slightly increased classification ambiguity. Fake images are correctly identified with a recall of approximately 98.8%, whereas real images achieve a recall of approximately 96.5%. This mild asymmetry reflects the intrinsic difficulty of large-scale real–fake detection, where authentic samples often lack distinctive manipulation artifacts. Nevertheless, the confusion matrix maintains clear diagonal dominance, confirming reliable and unbiased classification behavior across a diverse and heterogeneous dataset.

**Table 7:** Overall performance comparison on big-scale datasets.

Dataset	Overall Accuracy	Weighted Accuracy	Macro Precision	Macro Recall	Macro F1	Weighted F1	Micro F1	Global Kappa	BER	G-Mean	95% Confidence Interval
Real vs. AI-Generated Faces	99.12%	99.12%	0.9909	0.9910	0.9909	0.9912	0.9912	0.9819	0.0090	0.9910	[0.9901, 0.9921]
130 K Real vs. Fake Face	97.59%	97.59%	0.9756	0.9765	0.9759	0.9759	0.9759	0.9517	0.0235	0.9764	[0.9743, 0.9773]

**Table 8:** Class-wise performance metrics on big-scale datasets.

Dataset	Class	Accuracy	Precision	Recall	Specificity	F1-score	AUC	Error Rate	Balanced Acc	Kappa	G-Mean	Youden’s J
Real vs. AI-Generated Faces	Fake	0.98999	0.98902	0.98999	0.99200	0.98951	0.99100	0.010009	0.99100	0.98273	0.99099	0.98199
	Real	0.99200	0.99271	0.99200	0.98999	0.99235	0.99100	0.00800	0.99100	0.98273	0.99099	0.98199
	Average	0.99100	0.99086	0.99100	0.99100	0.99093	0.99100	0.009005	0.99100	0.98273	0.99099	0.98199
130 K Real vs. Fake Face	Fake	0.98836	0.96203	0.98836	0.96457	0.97502	0.97647	0.011641	0.97647	0.95181	0.97639	0.95293
	Real	0.96457	0.98916	0.96457	0.98836	0.97671	0.97647	0.035429	0.97647	0.95181	0.97639	0.95293
	Average	0.97647	0.97559	0.97647	0.97647	0.97586	0.97647	0.023535	0.97647	0.95181	0.97639	0.95293

#### Precision–recall and ROC curve analysis on big-scale datasets

The precision–recall (PR) curves for the Real vs. AI-Generated Faces dataset shown in Fig. 6b demonstrate near-perfect behavior across almost the entire recall range. The average precision (AP) reaches approximately 0.987 for AI-generated images and 0.992 for real images, indicating excellent precision retention even at high recall levels. The corresponding ROC curves in Fig. 6c achieve an AUC of approximately 0.991 for both classes, confirming near-ideal discriminative capability at scale.

For the 130 K Real vs. Fake Face dataset, the PR curves shown in Fig. 6e maintain high precision across most recall values, with AP values of approximately 0.959 for fake images and 0.980 for real images. A more noticeable precision degradation occurs only at extreme recall levels, reflecting increased ambiguity inherent in large-scale and heterogeneous real–fake data. The corresponding ROC curves in Fig. 6f achieve an AUC of approximately 0.976, confirming strong threshold-independent discrimination despite dataset complexity.

#### Comparative insights on big-scale datasets

A comparison between the two big-scale datasets highlights complementary characteristics. While Real vs. AI-Generated Faces benefits from more structured and consistent generative artifacts, leading to near-perfect performance, the 130 K Real vs. Fake Face dataset introduces greater variability and subtle manipulations, resulting in a modest but expected reduction in accuracy and agreement metrics. Importantly, the proposed model consistently maintains accuracy above 97%, balanced macro metrics, and strong statistical reliability across both datasets.

### Ablation study dataset and experimental protocol

The ablation experiments were conducted using the Real and Fake Face Detection dataset^65^, which contains a diverse collection of authentic and manipulated facial images. The dataset includes both expert-level Photoshop manipulations and deepfake-generated forgeries, covering a wide range of realistic facial alterations affecting regions such as the eyes, nose, mouth, and overall facial structure. These variations introduce substantial visual complexity and reflect realistic challenges encountered in deepfake face detection.

The dataset comprises 2,041 facial images, including 1,081 real and 960 fake samples, resulting in a near-balanced class distribution. Fake images include both localized region-level manipulations and full-face identity swaps generated using advanced deepfake techniques and professional image editing tools.

For the ablation study, a fixed 90–10% training–testing split was employed across all experiments. This controlled setup was designed to isolate and quantify the contribution of each pipeline component under identical conditions. All ablation experiments followed the same data partitioning strategy and experimental protocol to ensure consistency and fair comparison across different model configurations, enabling a focused evaluation of architectural and feature-design choices independent of dataset-scale effects examined in the cross-dataset generalization experiments. The ablation study systematically evaluates the contribution of each component of the proposed framework, with quantitative results summarized in Table 9.

#### End-to-End CNN baseline performance

The ablation study begins by evaluating end-to-end CNN architectures trained directly as classifiers, without feature decoupling or the use of external classifiers. This configuration represents a common baseline in many deepfake detection studies and provides insight into the discriminative capacity of standalone CNN representations. As reported in Table 9, the performance of these end-to-end models is relatively limited. ResNet-50 and MobileNet achieve accuracies of 69.60% and 69.11%, respectively, while ShuffleNet performs considerably worse, reaching only 62.27% accuracy with a notably low recall of 0.491.

Although these models demonstrate reasonable specificity, their reduced recall and AUC values indicate a high false-negative rate, particularly when detecting subtle or localized facial manipulations. This behavior highlights a key limitation of end-to-end CNN training: the learned representations tend to emphasize high-level semantic content while failing to consistently capture fine-grained forensic artifacts. The results suggest that relying solely on end-to-end CNN classifiers is insufficient for robust deepfake face detection, especially under limited training data or subtle manipulation scenarios, motivating the need for architectural diversity, feature fusion, and decoupled classification strategies.

**Table 9:** Performance evaluation of the proposed approach.

Methods	Feature vector size			Accuracy	Training Time	AUC	Recall	Specificity	Precision	F-Measure
*Resnet50*	*-*			69.6	16 min 11 s	69.68	0.685	0.708	0.725	0.704
*Shufflenet*	*-*			62.27	8 min 12 s	62.04	0.491	0.75	0.688	0.573
*MobileNet*	*-*			69.11	11 min 55 s	69.33	0.657	0.729	0.732	0.692
ResNet50	2048	Linear		89.71%	16.257	0.9569	0.898	0.896	0.907	0.902
		Quadratic		90.69%	10.685	0.9604	0.917	0.896	0.908	0.912
		Cubic		89.22%	18.026	0.9529	0.898	0.885	0.898	0.898
		Gaussian		90.20%	22.758	0.954	0.917	0.885	0.9	0.908
ShuffleNet	544	Linear		89.71%	1.7986	0.9409	0.889	0.906	0.914	0.901
		Quadratic		90.20%	1.827	0.9415	0.889	0.917	0.923	0.906
		Cubic		89.22%	2.5525	0.9452	0.889	0.896	0.906	0.897
		Gaussian		89.22%	1.4191	0.9393	0.889	0.896	0.906	0.897
MobileNet	4096	Linear		87.25%	3.8691	0.9387	0.889	0.854	0.873	0.881
		Quadratic		88.73%	6.9811	0.9341	0.889	0.885	0.897	0.893
		Cubic		87.75%	5.4911	0.9375	0.88	0.875	0.888	0.884
		Gaussian		88.24%	6.4714	0.9363	0.917	0.844	0.868	0.892
mobile_res50	3328	Linear		92.65%	21.402	0.9798	0.926	0.927	0.935	0.93
		Quadratic		95.59%	16.377	0.9821	0.954	0.958	0.963	0.958
		Cubic		94.61%	21.117	0.9808	0.954	0.938	0.945	0.949
		Gaussian		95.10%	30.803	0.9828	0.954	0.948	0.954	0.954
mobile_shuffle	1824	Linear		95.10%	7.4608	0.9934	0.935	0.969	0.971	0.953
		Quadratic		95.59%	11.162	0.9933	0.944	0.969	0.971	0.957
		Cubic		95.10%	10.527	0.9932	0.935	0.969	0.971	0.953
		Gaussian		95.10%	15.109	0.9902	0.944	0.958	0.962	0.953
res50_shuffle	2592	Linear		92.65%	11.245	0.9797	0.963	0.885	0.904	0.933
		Quadratic		92.65%	15.309	0.9778	0.963	0.885	0.904	0.933
		Cubic		92.65%	15.411	0.9781	0.963	0.885	0.904	0.933
		Gaussian		92.16%	23.864	0.9776	0.963	0.875	0.897	0.929
BOVW-Based HOG	500	Linear		79.41%	4.1524	0.8404	0.824	0.76	0.795	0.809
		Quadratic		79.90%	4.5068	0.8544	0.841	0.76	0.796	0.818
		Cubic		79.90%	3.6668	0.8658	0.843	0.75	0.791	0.816
		Gaussian		79.41%	5.5972	0.8523	0.843	0.74	0.784	0.812
Fused CNN	3872	Linear		95.10%	22.172	0.9892	0.954	0.948	0.954	0.954
		Quadratic		95.59%	21.849	0.9903	0.954	0.958	0.963	0.958
		Cubic		95.59%	17.868	0.9911	0.972	0.938	0.946	0.959
		Gaussian		95.10%	35.313	0.9924	0.972	0.927	0.938	0.955
Proposed	4372	Linear		96.57%	23.018	0.9945	0.972	0.958	0.963	0.967
		Quadratic		97.55%	25.999	0.9942	0.991	0.958	0.964	0.977
		Cubic		96.08%	28.335	0.9945	0.963	0.958	0.963	0.963
		Gaussian		96.08%	44.573	0.9944	0.981	0.938	0.946	0.963

#### Justification of choosing CNN Backbones

Building on the limitations observed in the end-to-end CNN baseline results, the selection of CNN backbones in this work was guided by the need to balance representational strength, architectural diversity, and computational efficiency. Instead of relying on a single deep network, the proposed framework integrates three heterogeneous CNN architectures, ResNet-50, MobileNet, and ShuffleNet, to reduce architectural bias and improve robustness to variations in facial appearance and manipulation patterns.

As shown in the end-to-end baseline evaluation on the Real and Fake Face Detection dataset^65^ using a consistent 90–10% split, these three models demonstrate the strongest performance among the CNN architectures considered. Specifically, ResNet-50 and MobileNet achieve accuracies of 69.60% and 69.11%, respectively, while ShuffleNet achieves 62.27%, all clearly exceeding chance-level classification. These results identify them as the most suitable baseline CNN backbones for further feature fusion within the proposed framework.

For additional context, Table 10 presents indicative baseline accuracies for several widely used pre-trained CNN architectures evaluated under the same end-to-end training configuration and hyperparameters applied to ResNet-50, MobileNet, and ShuffleNet, ensuring fair comparison. Under identical training conditions, these alternative models exhibit more limited performance, generally within the 56–62% accuracy range. AlexNet achieves 56.5% accuracy, and VGG16 reaches 58.0%, reflecting limitations associated with shallow feature representations or excessive parameter redundancy and memory demands. More recent architectures such as Inception V3 (60.5%), DenseNet201 (61.0%), and EfficientNet-B0 (59.2%) show slightly improved accuracy; however, their increased architectural complexity, feature redundancy, or sensitivity to hyperparameter tuning reduces their suitability for a stable and scalable hybrid forensic framework.

In contrast, the selected CNN backbones represent complementary architectural design philosophies. ResNet-50 contributes strong high-level semantic representations through residual learning, MobileNet enables efficient extraction of discriminative mid-level features using depthwise separable convolutions, and ShuffleNet provides lightweight and diverse representations via group convolutions and channel shuffling. Together, these models offer a balanced combination of semantic depth, efficiency, and architectural diversity, making them well suited for robust, interpretable, and computationally efficient deepfake face detection within the proposed hybrid framework.

**Table 10:** Comparison of CNN models.

Model	Model size (MB)	Depth/Design	Computational cost	Key limitation for hybrid framework	Indicative accuracy (%)
ResNet-50^16^	98	50-layer residual	Moderate	Higher cost, but strong semantics	69.60
MobileNet^17^	~ 14	Depthwise separable	Very low	Lower semantic depth alone	69.11
ShuffleNet^18^	5.5	Group conv + shuffle	Very low	Compact, limited alone	62.27
AlexNet^66^	233	8 layers	Very high	Shallow features; outdated design	56.5
VGG-16^67^	528	16 layers	Extremely high	Excessive memory & redundancy	58.0
Inception-V3^68^	91	Multi-branch	High	Complex fusion & interpretability	60.5
DenseNet-201^69^	77	201 layers	High	Feature redundancy; memory overhead	61.0
EfficientNet-B0^70^	20	Compound scaling	Moderate	Sensitive to tuning and scaling	59.2

#### Impact of SVM classification on single-CNN features

To improve discriminative performance, deep feature vectors extracted from individual CNN backbones were classified using SVMs with different kernel functions. As shown in Table 9, this modification leads to a substantial performance improvement, with accuracies increasing to approximately 89–91% across CNN architectures. Among the evaluated kernels, the quadratic kernel consistently yields the best results, indicating moderately nonlinear decision boundaries in the deep feature space. Notably, ShuffleNet achieves competitive accuracy with significantly reduced training time, highlighting its computational efficiency. However, performance remains bounded below 91%, indicating that single-CNN representations still lack complementary forensic cues.

#### Effect of multi-CNN feature fusion

To capture complementary semantic representations, feature-level fusion was performed by combining deep features from multiple CNN backbones. The results in Table 9 demonstrate that multi-CNN fusion consistently outperforms single-CNN feature representations. Fused configurations such as MobileNet + ResNet-50 and MobileNet + ShuffleNet achieve accuracies exceeding 95%, confirming the effectiveness of integrating heterogeneous deep features. The quadratic SVM kernel again provides the most stable and accurate performance. In particular, the MobileNet + ShuffleNet fusion achieves high accuracy with a compact feature vector and reduced training time, offering a favorable accuracy–complexity trade-off. The fully fused CNN representation further stabilizes performance, achieving AUC values above 0.99.

#### Handcrafted BoVW–HOG feature analysis

The standalone effectiveness of handcrafted forensic descriptors was evaluated using BoVW-based HOG features with different SVM kernels. As summarized in Table 9, handcrafted features achieve moderate detection performance, with accuracies in the range of 79–80%. While the quadratic kernel yields slightly improved AUC and F-measure values, handcrafted descriptors remain inferior to deep representations. Nevertheless, their very low training time highlights their computational efficiency and confirms their ability to capture useful local texture and gradient inconsistencies.

#### Proposed hybrid model: deep and handcrafted integration

The final ablation evaluates the proposed hybrid framework, which integrates fused multi-CNN deep features with handcrafted BoVW–HOG descriptors and performs classification using optimized SVM kernels. As evidenced in Table 9, this hybrid integration yields the best overall performance. Using the quadratic kernel, the proposed model achieves an accuracy of 97.55%, an F-measure of 0.977, and an AUC exceeding 0.99. The high recall (0.991) confirms strong sensitivity to fake images, while the maintained specificity (0.958) ensures reliable identification of genuine samples. Although Gaussian kernels provide comparable accuracy, their significantly higher training time makes the quadratic kernel the most practical choice.

#### Ablation study summary

Overall, the ablation results summarized in Table 9 reveal a clear and consistent performance progression. End-to-end CNN models provide limited discrimination, SVM-based classification substantially improves performance, multi-CNN fusion enhances robustness, and handcrafted features contribute complementary low-level forensic cues. The proposed hybrid framework effectively integrates these components, achieving the highest accuracy, strongest generalization, and best balance between performance and computational efficiency.

#### Rationale for fusing handcrafted and deep learning features

##### Fusion of handcrafted and deep learning features

The fusion of handcrafted and deep learning features in the proposed framework is motivated by the need to balance detection accuracy with reliability, particularly in high-stakes applications such as digital forensics and legal investigations. While deep CNN features are effective at modeling complex semantic inconsistencies, end-to-end CNN classifiers in our baseline experiments achieve only 62.27–69.60% accuracy and exhibit low recall in certain cases (e.g., 0.491), indicating a high risk of false negatives. Such behavior limits the reliability of deep-only models in forensic scenarios, where missed detections can have serious legal and investigative consequences.

In contrast, handcrafted BoVW–HOG descriptors encode explicit and well-understood local image characteristics, such as gradient orientations and texture distributions, which are widely used in classical image forensics. When evaluated independently using an SVM classifier, these features achieve moderate yet stable performance (approximately 79–80% accuracy), providing reliable forensic evidence. Moreover, handcrafted features offer consistent and interpretable representations that are less sensitive to dataset bias and manipulation-specific artifacts. This indicates that deep and handcrafted features capture different aspects of the data, and their integration improves detection reliability and robustness. Similar benefits of hybrid representations have been observed in our prior works across medical imaging^44^, agriculture^54^, and industrial inspection tasks^46^.

##### Model Interpretability and Transparent Decision-Making

Beyond performance improvements, the fusion strategy enhances model interpretability, which is critical in legal contexts. Handcrafted BoVW–HOG features provide transparent and reproducible representations linked to localized visual artifacts, enabling forensic experts to understand and justify model decisions. These features allow tracing predictions to specific gradient and texture inconsistencies that can be visually inspected and validated.

The hybrid framework achieves 97.55% accuracy, with an AUC of 0.994 and recall of 0.991, confirming that the integration of handcrafted and deep features improves overall detection capability. Furthermore, the use of an SVM classifier strengthens interpretability by learning explicit, margin-based decision boundaries instead of relying on opaque end-to-end deep models. This design provides clearer insight into the decision process and reduces model opacity. Therefore, the proposed fusion strategy is not only a performance-driven choice but a necessary design for achieving reliable, transparent, and legally defensible deepfake detection.

#### Overfitting mitigation and generalization strategy

Although the proposed framework integrates multiple CNN backbones and handcrafted features, it is intentionally not designed as an end-to-end deep learning model. Instead, all CNNs are used strictly as feature extractors, while classification is decoupled from representation learning through a margin-based Support Vector Machine (SVM). This architectural choice reduces model capacity at the decision stage and mitigates overfitting commonly observed in deep classifiers. Unlike fully connected neural classifiers, SVMs enforce maximum margin separation, which promotes better generalization in high-dimensional and fused feature spaces. Consequently, this decoupled design directly limits overfitting by controlling model complexity.

Several explicit and implicit regularization strategies are incorporated throughout the framework. Transfer learning is applied with limited fine-tuning depth and a small number of epochs (≤ 10), preventing excessive parameter adaptation and reducing sensitivity to dataset-specific artifacts. Early stopping based on validation performance is employed for all CNN models to further control overfitting. In addition, data augmentation techniques, including horizontal flipping, rotation, and contrast variation, are used to increase training diversity and improve robustness to variations in pose, illumination, and facial appearance.

Within the handcrafted feature branch, overfitting is further mitigated by retaining only the strongest 20% of detected keypoints, thereby suppressing noisy or weak local responses that may lead to unstable representations. A fixed visual vocabulary size is used in the BoVW construction, and histogram normalization ensures scale-invariant and comparable feature representations across images. At the fusion stage, feature-level concatenation followed by SVM classification is adopted instead of deep interaction or attention-based fusion modules, which are more prone to feature co-adaptation and excessive complexity. This design supports stable learning behavior and improved generalization.

In addition to standard validation strategies, generalization is further evaluated through extensive cross-dataset testing. The proposed model is assessed on six heterogeneous datasets that vary significantly in scale, manipulation type, facial visibility, and image quality. Consistent performance across small-, medium-, and large-scale benchmarks, exceeding 97% accuracy in all medium- and large-scale datasets, provides strong empirical evidence of cross-dataset generalization. Such evaluation is more representative of real-world robustness than k-fold validation within a single dataset, which may suffer from optimistic bias due to shared data distributions.

Finally, to ensure reliable component-level analysis, a controlled ablation study is conducted using fixed training–testing splits across all configurations. This approach avoids performance inflation caused by repeated data reuse in cross-validation while enabling precise attribution of performance gains to individual architectural and feature design choices.

### Comparison with state-of-the-art methods

This subsection compares the proposed hybrid deepfake detection framework with representative state-of-the-art methods reported in the literature. For a fair and consistent evaluation, all methods listed in Table 11 were evaluated using a 90–10% training–testing split, following the experimental protocol adopted in the original studies or reimplemented under identical conditions where applicable.

As shown in Table 11, early CNN-based approaches exhibit limited discriminative capability for deepfake face detection. In particular, the VGG16-based model^4^ records the lowest performance, achieving only 52.37% accuracy and 56.68% F1-score, indicating weak generalization and poor sensitivity to manipulation artifacts. The ResNet-50 model^4^ improves performance marginally, achieving 58.24% accuracy and 62.05% specificity, while the conventional CNN model^4^ shows similar accuracy but suffers from lower specificity and F1-score, reflecting an increased false-positive rate.

A notable improvement is observed with the fused CNN+ResNet50 + VGG16 model^4^, which raises accuracy to 75.79% and recall to 77.43%, demonstrating the benefit of multi-network feature aggregation. However, despite this improvement, the performance remains insufficient for reliable real-world deepfake detection.

More recent methods further enhance performance. Meena et al.^71^ propose a few-shot transfer learning framework (FSTL-SA) and achieve approximately 82.40% accuracy, highlighting the effectiveness of hybrid deep representations for facial analysis tasks. Similarly, the methods reported in^72,73^ attain accuracies of 82.98% and 77.0%, respectively, but still fall short in achieving consistently high precision and recall simultaneously.

In contrast, the proposed hybrid method significantly outperforms all compared approaches, achieving an accuracy of 97.55%, a specificity of 95.8%, and an F1-score of 97.7%. The high recall of 99.1% indicates exceptional sensitivity to fake samples, while the strong precision (96.4%) confirms reliable identification with minimal false positives. These results demonstrate that the proposed framework achieves near-perfect classification performance under the same 90:10 evaluation protocol.

Overall, the comparative analysis confirms that integrating fused multi-CNN deep features with handcrafted forensic descriptors yields a substantial performance gain over both traditional CNN-based models and recent hybrid approaches. The achieved accuracy close to 98%, together with balanced precision–recall behavior, highlights the robustness and practical applicability of the proposed method for real-world deepfake face detection scenarios.

**Table 11:** Comparison of the proposed method with state-of-the-art deepfake detection approaches (90–10% Split).

Model	Accuracy (%)	Specificity (%)	F1-Score (%)	Precision (%)	Recall (%)
VGG16^4^	52.37	57.85	56.68	55.73	54.35
ResNet50^4^	58.24	62.05	61.13	62.11	62.37
CNN^4^	58.22	53.95	53.65	52.34	52.95
CNN + ResNet50 + VGG16 ^4^	75.79	77.91	75.68	73.58	77.43
Ref.^72^	82.98	88.24	83.45	79.16	88.24
Ref.^73^	77.00	73.00	–	79.00	–
Ref.^71^	82.40	–	–	–	–
Proposed Hybrid Method	97.55	95.80	97.70	96.40	99.10

### Computational complexity analysis

Let $\:N$ denote the number of images in the dataset, $\:P$ the average number of retained keypoints per image, $\:D$ the dimensionality of the HOG descriptor, $\:K$ the number of visual words in the BoVW vocabulary, $\:F$ the dimensionality of the fused feature vector, and $\:{C}_{R},{C}_{M},{C}_{S}$ the per-image forward-pass costs of ResNet50, MobileNet, and ShuffleNet, respectively. To clearly analyze computational overhead, we explicitly distinguish between offline training complexity and online inference complexity.

#### Offline training complexity

The following operations are executed only once during the training phase and do not affect deployment time performance.

*Handcrafted Feature Extraction (Training Data)*: Local forensic features are extracted by detecting interest points using SURF, FAST, and BRISK, followed by HOG descriptor computation. The complexity scales as: $\:O(N\cdot\:P\cdot\:D)$.

In practice, this cost is reduced because only the strongest 20% of detected keypoints are retained, significantly constraining $\:P$.

*BoVW Visual Vocabulary Construction*: HOG descriptors are clustered using K-means + + with complexity: $\:O(T\cdot\:K\cdot\:P\cdot\:D)$

where $\:T$ denotes the number of clustering iterations. This step is performed once offline, and the cost remains bounded because the vocabulary size is fixed $\:(K=500)$.

*CNN Fine-Tuning* Fine-tuning the CNN backbones (ResNet-50, MobileNet, ShuffleNet) is performed offline using transfer learning and a limited number of epochs $\:(\le\:10).$ Although computationally intensive, this cost is amortized over deployment and does not impact inference.

*SVM Training* Training the SVM classifier has a worst-case complexity: $\:O\left({N}^{2}\cdot\:F\right).$

This step is also strictly offline and is mitigated in practice through moderate dataset size and optimized solvers.

#### Online inference complexity

During deployment, the proposed framework requires only lightweight and deterministic operations per test image. The computational complexity of each stage is given as follows:

- The complexity of keypoint-based HOG feature extraction is given by $\:O(P\cdot\:D)$.
- The complexity of CNN forward passes is given by $\:O\left({C}_{R}+{C}_{M}+{C}_{S}\right)$, where MobileNet and ShuffleNet ensure low computational cost, while ResNet-50 provides complementary semantic representations.
- The complexity of BoVW histogram encoding is given by $\:O(P\cdot\:K)$, which is further accelerated using approximate nearest-neighbor search techniques.
- The complexity of feature-level fusion is given by $\:O\left(F\right)$.
- The complexity of SVM prediction is given by $\:O\left(F\right)$.

#### Overall complexity and performance trade-off

Although the proposed hybrid framework integrates multiple components, it is not computationally prohibitive in practice due to its clear offline–online separation and feature-centric design. All computationally expensive operations are confined to the offline training phase, while the online inference complexity remains linear with respect to feature dimensionality and keypoint count.

At inference time, the pipeline consists of keypoint-based HOG extraction, forward passes through lightweight CNN backbones, feature concatenation, and a single SVM prediction step. The SVM classifier has a complexity of $\:O\left(F\right)$, resulting in negligible runtime overhead. Compared to end-to-end deep or transformer-based models that require iterative or attention-based computation, the proposed framework enables predictable and low-latency inference.

The ablation study (Sect. 4.6, Table 9) provides explicit evidence of the performance–cost trade-off. Lightweight configurations such as MobileNet + ShuffleNet achieve high detection accuracy (approximately 95.6%) with lower computational overhead, while the full hybrid configuration reaches 97.55% accuracy with only a moderate increase in feature dimensionality and training cost. This demonstrates that performance improvements are incremental and controlled rather than resulting from excessive model complexity.

Furthermore, the selection of MobileNet and ShuffleNet ensures computational efficiency due to their low parameter counts and reduced floating-point operations, while ResNet-50 contributes complementary semantic information without dominating the computational profile. The framework also operates strictly at the image level, avoiding the high computational cost associated with video-based methods that require temporal modeling, frame alignment, or sequential inference.

Overall, the proposed framework achieves a controlled and practical trade-off between computational cost and detection performance. By confining expensive operations to the training phase, leveraging lightweight architectures, and employing efficient classification, the model enables robust, interpretable, and near real-time deepfake detection suitable for real-world image-level deployment.

### Generalization, real-world deployment, and real-time considerations

#### Cross-dataset generalization analysis

Although the proposed framework is evaluated on benchmark datasets, the experimental results provide strong evidence of its ability to generalize across diverse and realistic conditions. The six datasets used in this study vary significantly in scale, manipulation type, image quality, and facial visibility. These include large-scale datasets (130 K Real vs. Fake Face, Real vs. AI-Generated Faces), medium-scale datasets with limited or cropped facial context (Real/Fake Cropped Faces, RVF10K), and small-scale challenging datasets containing visually ambiguous samples (Fake vs. Real Faces (Hard), Human Faces). The model consistently maintains high performance across these heterogeneous datasets, achieving accuracies above 97% on all medium- and large-scale benchmarks and above 97.6% even on visually challenging small-scale datasets. This consistent behavior provides strong empirical evidence of robustness to dataset variability and realistic operating conditions.

Further support for generalization is demonstrated through the ablation study. As shown in Table 9, end-to-end CNN classifiers achieve limited performance (approximately 62–70% accuracy), indicating sensitivity to dataset-specific patterns. When deep features are decoupled from classification and combined with an SVM classifier, performance improves significantly (approximately 90% accuracy). The fusion of multiple CNN backbones further increases accuracy to over 95%, demonstrating that architectural diversity reduces reliance on a single representation bias. The highest performance is achieved when handcrafted BoVW–HOG features are integrated with multi-CNN features, reaching 97.55% accuracy, an AUC of 0.99, and high recall (0.991). This progressive improvement confirms that each component contributes complementary information that enhances generalization.

From a design perspective, this robustness can be attributed to the hybrid forensic-first architecture. Handcrafted BoVW–HOG descriptors capture stable local gradient and texture characteristics that are less sensitive to variations in dataset distribution or manipulation techniques. In parallel, the use of heterogeneous CNN backbones (ResNet-50, MobileNet, and ShuffleNet) enables the extraction of complementary semantic features, balancing representational strength and efficiency. The SVM classifier further enhances generalization by enforcing stable, margin-based decision boundaries, reducing overfitting compared to fully end-to-end deep classifiers.

Despite these strengths, it is acknowledged that complete real-world generalization cannot be fully guaranteed without evaluation on entirely unseen datasets, open-set scenarios, and temporal modeling for video-based deepfakes. These aspects are beyond the scope of the current image-level study and are identified as important directions for future work. Nevertheless, the consistent performance across multiple heterogeneous datasets, supported by ablation analysis, demonstrates that the proposed framework provides a robust and generalizable solution for practical image-based deepfake detection.

#### Real-world deployment and practical applicability

Beyond cross-dataset generalization, practical deployment considerations are critical for real-world applicability. Many forensic and security applications operate primarily on still images rather than fully annotated video streams. Typical use cases include social media profile verification, identity document authentication, biometric access control, online media analysis, and forensic investigation of disputed visual evidence. The image-level design of the proposed framework aligns naturally with these scenarios, where only individual frames or isolated facial images are available, and where reliable, interpretable, and computationally efficient detection is required. In practice, this enables a straightforward deployment pipeline in which input images are processed independently, followed by direct classification, ensuring scalable and low-latency operation.

Although the framework does not explicitly model temporal dynamics, it can be effectively applied in video-based environments through frame-level analysis. In practical video pipelines, the model can be applied to selected key frames or detected face tracks, and predictions can be aggregated using lightweight strategies such as majority voting, confidence averaging, or threshold-based scoring to produce video-level decisions. This approach enables seamless integration into existing video processing systems without requiring additional training or architectural modifications. Moreover, it avoids the significant computational cost, data requirements, and instability associated with temporal deep models such as recurrent networks, 3D convolutional architectures, or transformer-based methods.

In the summary, the proposed framework achieves a practical balance between accuracy, efficiency, and interpretability, making it well suited for deployment in real-world forensic and security applications involving both static images and video streams. Therefore, the method is not limited to benchmark evaluation but is directly applicable to real-world scenarios requiring reliable and scalable deepfake detection.

#### Comparison with real-time deepfake detection methods

Recent advancements in deepfake detection have increasingly focused on real-time analysis, particularly in video-based and multimodal settings. Several studies have made significant contributions by leveraging temporal, physiological, and cross-modal cues for detecting manipulated content under streaming conditions. In particular, the work of Javed et al.^74,75^ presents transformer-based and hybrid deep learning frameworks that utilize gaze dynamics, blink patterns, and eye-movement behavior, demonstrating strong effectiveness for real-time deepfake video detection. These studies provide valuable insights into the use of subtle physiological signals as reliable indicators of facial manipulation in continuous video streams.

Furthermore, Javed et al.^76^ extend this direction by incorporating audio–visual synchronization and lip movement consistency, enabling robust real-time detection through multimodal temporal alignment. More recent work^77^ explores the integration of local–global feature reasoning and diffusion-based representations, further improving detection performance in complex real-time environments. Collectively, these contributions highlight the growing maturity and practical relevance of multimodal and temporal modeling approaches in real-time deepfake detection systems.

In addition to deepfake-specific research, related studies by Dahri et al.^78,79^ demonstrate the effectiveness of efficient deep learning architectures for real-time vision tasks, emphasizing the importance of optimized model design for deployment in time-critical environments. Table 12 presents a high-level comparison between representative real-time deepfake detection approaches and the proposed method.

**Table 12:** Comparison with recent real-time deepfake detection approaches.

Category	Representative work	Key approach	Strengths	Relation to proposed work
Physiological Signal-Based	Javed et al.^74,75^	Gaze, blink, and eye-movement analysis with transformer/hybrid models	Effective real-time detection using temporal physiological cues	Complementary; proposed method targets scenarios without temporal signals
Multimodal Temporal Analysis	Javed et al.^76^	Audio–visual synchronization and lip movement consistency	Robust detection using cross-modal alignment	Complementary; proposed method operates on single images without audio/temporal data
Generative & Multimodal Models	Javed et al.^77^	Local–global feature integration with diffusion models	Strong performance in complex real-time environments	Complementary; proposed method emphasizes interpretability and cross-dataset robustness
Real-Time Vision Systems	Dahri et al.^78,79^	Efficient CNN and YOLO-based architectures	Demonstrates high-efficiency inference in time-critical applications	Supports efficiency-driven design of the proposed hybrid framework
Proposed Method	This Work	Hybrid BoVW–HOG + multi-CNN + SVM	High accuracy, interpretability, cross-dataset generalization	Complements real-time video methods by addressing image-level forensic scenarios

Building on these important advances, the proposed framework addresses a complementary scenario focused on image-level forensic analysis, where temporal or multimodal information may not be available. Many real-world applications, such as identity verification, forensic evidence analysis, and social media authentication, rely on single images rather than continuous video streams. In such cases, the proposed hybrid framework emphasizes robustness, interpretability, and cross-dataset generalization.

Moreover, the proposed approach remains compatible with real-time video pipelines through frame-level analysis, where predictions can be aggregated using lightweight strategies such as majority voting or confidence averaging. Therefore, the proposed method should be viewed as complementary to existing real-time detection frameworks, extending deepfake detection capabilities to both static image and dynamic video scenarios.

### Conclusion and future work

This paper presented a robust and interpretable hybrid deepfake face detection framework that integrates handcrafted forensic descriptors with deep semantic representations to address the growing challenges posed by increasingly realistic face manipulation technologies. Unlike purely deep learning–based detectors, the proposed method explicitly combines BoVW representations built on HOG with feature-level fusion of multiple pre-trained CNN architectures, namely ResNet-50, MobileNet, and ShuffleNet. This design enables the model to jointly capture fine-grained local texture inconsistencies and high-level semantic manipulation cues.

Extensive experiments conducted across six benchmark datasets of varying scale and difficulty—including small-, medium-, and large-scale real–fake and AI-generated face datasets—demonstrate the effectiveness, robustness, and scalability of the proposed approach. The framework consistently achieves high accuracy (up to 99.12%), strong agreement beyond chance (Global Kappa > 0.95), low Balanced Error Rates, and stable macro-averaged performance, confirming balanced behavior across classes and datasets. In particular, the results on challenging small-scale and large-scale benchmarks highlight the model’s strong generalization capability under data scarcity, visual ambiguity, and realistic manipulation diversity.

The ablation study further validates the central design choices of the framework. While end-to-end CNN classifiers show limited discriminative power, the introduction of SVM-based classification substantially improves performance. Multi-CNN feature fusion enhances robustness by capturing complementary representations, and the integration of handcrafted BoVW–HOG features provides additional low-level forensic cues that significantly boost detection accuracy. The final hybrid model achieves the best overall performance, confirming that combining interpretable handcrafted features with deep semantic representations yields a more reliable and generalizable deepfake detection system.

Overall, the proposed framework offers a lightweight, accurate, and interpretable solution for image-only deepfake face detection, making it well suited for real-world forensic applications such as social media monitoring, identity verification, digital forensics, and content authenticity assessment.

Future research will extend the proposed hybrid framework beyond image-level analysis to video-based deepfake detection by incorporating temporal consistency modeling. Integrating lightweight temporal descriptors with the current handcrafted–deep feature fusion strategy may further enhance robustness against frame-level and motion-based manipulation artifacts commonly observed in deepfake videos.

Another important direction involves improving adaptability to unseen and evolving manipulation techniques. Future work will explore open-set and continual learning strategies that allow the model to detect previously unknown deepfake patterns without requiring complete retraining, thereby improving long-term reliability in real-world forensic applications.

The integration of additional handcrafted descriptors, particularly lightweight frequency-domain features, also represents a promising extension. Combining spatial gradient-based features with complementary frequency cues may further strengthen robustness against compression, resizing, and post-processing operations while maintaining interpretability and low computational cost.

Finally, future efforts will focus on optimizing the framework for real-time and edge deployment. Feature selection, dimensionality reduction, and model compression techniques will be investigated to reduce computational overhead and enable efficient deployment in resource-constrained environments such as mobile devices and online content moderation systems.

## Data Availability

The data that support the findings of this study are publicly available from Kaggle repositories, as cited in References^20–25^. These datasets are accessible for research use under the respective platform terms and conditions. No proprietary or confidential data were used in this study.
